# Combined Effects of Hybrid Pressurized Irrigation and High-Intensity Legume Intercropping on Productivity, Water Use Efficiency, and Soil Carbon Dynamics in Sugarcane-Based Cropping Systems

**DOI:** 10.3390/plants15193045

**Published:** 2026-10-05

**Authors:** Navnit Kumar, Shivani Ranjan, Vipin Kumar, Ravish Chandra, Santosh Kumar Thakur, Lalita Rana, Sumit Sow

**Affiliations:** 1 Department of Agronomy, Sugarcane Research Institute, Dr. Rajendra Prasad Central Agricultural University, Pusa 848 125, Bihar, India; 2 Department of Agronomy, Tirhut College of Agriculture, Dholi, Dr. Rajendra Prasad Central Agricultural University, Pusa 848 125, Bihar, India; 3 Department of Soil Science, Post Graduate College of Agriculture, Dr. Rajendra Prasad Central Agricultural University, Pusa 848 125, Bihar, Indiasumitsow19@gmail.com (S.S.); 4 Department of Irrigation & Drainage Engineering, College of Agricultural Engineering and Technology, Dr. Rajendra Prasad Central Agricultural University, Pusa 848 125, Bihar, India; 5 Directorate of Research, Dr. Rajendra Prasad Central Agricultural University, Pusa 848 125, Bihar, India

**Keywords:** drip irrigation, cane equivalent yield, carbon sequestration, profitability, soil health, resource-use efficiency

## Abstract

To evaluate the combined effects of hybrid pressurized irrigation (drip irrigation for sugarcane and micro-sprinkler irrigation for intercrops), legume-based intercropping, and residue incorporation on crop productivity, water use efficiency, soil organic carbon dynamics, and carbon sequestration in a sugarcane-ratoon I-ratoon II production system. A three-year field experiment (2017–2020) was conducted at RPCAU, Pusa, Bihar, India. Eight treatments involving different irrigation methods, intercropping systems, and residue management practices were evaluated in a randomized block design with three replications. All parameters were assessed across the plant crop, ratoon-I, and ratoon-II. Drip-irrigated sole sugarcane produced the highest cane yield (107.3, 102.1 and 98.4 t ha^−1^) in plant, ratoon I and ratoon II, compared with surface irrigation, respectively. The highest cane equivalent yield (154.0–159.1 t ha^−1^) and soil organic carbon stock (33.6 Mg ha^−1^) were recorded under hybrid pressurized irrigation + French bean–green gram intercropping + residue incorporation. Drip irrigation improved water use efficiency by 92% (1529 kg ha^−1^ cm^−1^). After three crop cycles, T8 recorded maximum total soil organic carbon (17.81 g kg^−1^), particulate organic matter (3.62 g kg^−1^), soil microbial biomass carbon (292.5 mg kg^−1^), carbon pool index (1.52), carbon lability index (1.54), carbon management index (234) and soil organic carbon stock (33.6 Mg ha^−1^), indicating improved soil carbon accumulation and sequestration. Integration of hybrid pressurized irrigation, legume intercropping, and residue incorporation improved productivity, water use efficiency, profitability, soil health, and carbon sequestration, and T8 treatment emerged as the most sustainable option for enhancing resource-use efficiency and long-term soil carbon storage in sugarcane-based cropping systems.

## 1. Introduction

Sugarcane (*Saccharum officinarum* L.) is one of the most important commercial crops cultivated in tropical and subtropical regions of the world, contributing significantly to global sugar production, bioenergy generation and rural livelihoods [1]. Globally, sugarcane is grown on about 26 million hectares with an annual production exceeding 1.9 billion tonnes [2]. India is the second largest producer of sugarcane after Brazil, with nearly 5.8 million hectares under cultivation and a total production of around 490 million tonnes, contributing substantially to the national economy and supporting millions of farmers and workers associated with the sugar industry [2]. Despite its high yield potential and economic importance, sugarcane cultivation is often limited by inefficient utilization of natural resources, particularly irrigation water and soil nutrients, which poses serious challenges to sustainable crop production. Sugarcane is a long-duration crop occupying the field for nearly 10–12 months and is generally planted in wide rows, offering great opportunities for cultivation of short-duration intercrops during its initial growth period. Intercropping in sugarcane has been widely recognized as an efficient strategy to increase land use efficiency, diversify farm income and improve system productivity. Leguminous intercrops such as French bean (*Phaseolus vulgaris* L.) and green gram (*Vigna radiata* L.) are particularly beneficial when integrated with sugarcane, improving soil fertility and enhancing soil organic matter content. High-intensity intercropping in autumn-planted sugarcane is a type of intercropping system in which two short-duration intercrops are sequentially grown between sugarcane rows during the early growth stage of the main crop. Autumn sugarcane is in the field for a relatively longer period, and the wider inter-row space during the early stages of autumn sugarcane growth can be efficiently utilized for cultivating additional crops. Furthermore, the incorporation of legume residues into the soil further enriches soil organic carbon and promotes microbial activity, thereby improving soil health and long-term sustainability of the cropping system [3]. Intercropping also allows more efficient utilization of available resources such as sunlight, nutrients and moisture, resulting in increased productivity and profitability of the production system [4].

Water management is another crucial factor influencing the productivity and sustainability of sugarcane cultivation. Conventional surface irrigation methods, which are widely practiced in many sugarcane-growing regions, often lead to excessive water application, lower irrigation efficiency and higher energy consumption. Increasing pressure on water resources due to population growth, climate variability and competing demands from other sectors has made it crucial to adopt water-saving technologies in agriculture [5]. The adoption of pressurized irrigation systems such as drip irrigation and micro-sprinkler irrigation has gained considerable importance because of their ability to deliver water directly to the root zone of crops with reduced losses. These systems significantly improve irrigation efficiency, reduce water wastage and enhance water productivity compared with traditional irrigation methods. Moreover, pressurized irrigation systems facilitate precise application of water and nutrients, which ultimately improves crop growth, yield and resource-use efficiency. Furthermore, efficient irrigation combined with crop diversification and residue management can significantly influence soil carbon dynamics and soil biological processes [6]. Soil organic carbon plays a vital role in maintaining soil fertility, improving soil structure and enhancing nutrient cycling within agricultural systems. Higher soil organic carbon results in enhanced soil aggregation, better water-holding capacity and increased microbial activity. Integration of legumes and residue incorporation practices in sugarcane-based cropping systems can substantially enhance carbon inputs to the soil, thereby increasing soil carbon fractions and improving soil health over time [7]. Furthermore, enhancement of soil organic carbon through improved crop and irrigation management practices can significantly increase the carbon sequestration potential of agricultural soils. Therefore, adoption of sustainable sugarcane-based intercropping systems combined with efficient irrigation management may serve as an effective approach for enhancing soil carbon sequestration while simultaneously mitigating greenhouse gas emissions from agricultural systems. In addition to total soil organic carbon, assessment of carbon indices such as carbon pool index (CPI), carbon lability index (CLI) and carbon management index (CMI) provides a more comprehensive understanding of changes in both the quantity and quality of soil carbon under different management practices. These indices are sensitive indicators of soil health and sustainability, particularly under intensive cropping systems involving residue recycling and intercropping.

Although several studies have examined the benefits of intercropping or pressurized irrigation individually, limited information is available on the combined influence of high-intensity intercropping and hybrid pressurized irrigation systems on crop productivity, water-use efficiency, soil carbon dynamics and environmental sustainability in sugarcane-based cropping systems. A comprehensive understanding of these interactions is essential for developing efficient and climate-resilient production systems that optimize resource use while improving soil health. Based on the above research gaps, it was hypothesized that the integration of hybrid pressurized irrigation with high-intensity legume intercropping in a sugarcane-based cropping system could enhance crop productivity, improve water-use efficiency and soil carbon dynamics, while simultaneously increasing carbon sequestration, thereby improving the overall sustainability of the production system. Therefore, the present investigation was undertaken to evaluate different irrigation and intercropping combinations in order to identify an efficient management strategy for sugarcane cultivation that can enhance system productivity, improve soil health and reduce the overall environmental footprint of the production system.

## 2. Materials and Methods

### 2.1. Experimental Site

The field experiment was conducted during 2017–2018 to 2019–2020 at the research farm of Dr. Rajendra Prasad Central Agricultural University (RPCAU), Pusa, Bihar, India, which falls under the Middle Gangetic Plains Region (Agro-climatic Zone IV). The region is characterized by a sub-tropical climate with hot summers and cool winters. The average annual rainfall of the area is approximately 1100–1300 mm, most of which is received during the monsoon season (June to September) during 2017–2020, as indicated in Figure 1.

The experimental soil was sandy loam and slightly alkaline with moderate fertility status. The initial physico-chemical properties of soil at the start of the experiment were: sand 42.7%, silt 46.2%, clay 11.0%, pH 8.36, EC 0.33 dS m^−1^, field capacity 22.8%, bulk density 1.43 g cm^−3^, organic carbon 0.44%, available nitrogen 246 kg ha^−1^, available phosphorus 10.6 kg ha^−1^ and available potassium 102 kg ha^−1^.

### 2.2. Experimental Design and Treatment Details

The experiment was conducted for three consecutive crop cycles (plant crop, ratoon-I and ratoon-II) during 2017–2018 to 2019–2020 in a randomized block design with three replications. The study evaluated the combined effects of irrigation method, legume intercropping and residue incorporation on growth, productivity and resource-use efficiency of a sugarcane-based cropping system. Eight treatments were formulated by combining conventional surface irrigation and pressurized irrigation (drip and micro-sprinkler), with sole sugarcane and legume-based intercropping systems, with or without residue incorporation (Table 1 and Table 2). Hybrid pressurized irrigation consisted of drip irrigation applied to sugarcane rows and micro-sprinkler irrigation applied to intercrops.

### 2.3. Crop Establishment and Management Practices

Sugarcane variety ‘CoP 112’ was planted at 30: 120 cm (paired rows) row spacing, following recommended agronomic practices of the region. Healthy three-budded setts were used as planting material. Recommended spacing and crop management practices were followed throughout the experiment. Legume intercrops such as French bean and green gram were grown between sugarcane rows at 30 cm apart during early growth stages to utilize inter-row space efficiently and improve soil by enriching the soil with nutrients. Legume intercrops included French bean (*Phaseolus vulgaris* L.) and green gram (*Vigna radiata* L.), grown in three rows between sugarcane rows as depicted in Supplementary Table S1. Residues of green gram were incorporated into the soil after harvest in respective treatments. The seed rate in the intercropping system was distributed based on the area occupied by each crop, which is 82.5 and 18 kg/ha for French bean variety: Utkarsh and green gram variety: HUM-16, respectively. The recommended dose of N, P and K for sugarcane plant and ratoon crop was 150—37.1–49.8 and 170—21.8–49.8 kg/ha, respectively. Whereas for French bean and green gram, it was 100—21.8–24.9 and 20—19.6–16.6 kg/ha, respectively. Half of N and the entire dose of P and K to sugarcane, French bean and green gram were applied as basal. Remaining N was top-dressed in 2 equal splits at the initial and final stages of tillering in sugarcane and 30 and 60 days after sowing in French bean and green gram, as indicated in Supplementary Table S2. Standard agronomic practices, including weed management, plant protection measures and intercultural operations, were followed uniformly across treatments.

### 2.4. Irrigation Management

Irrigation was applied according to treatment specifications using surface irrigation, drip irrigation and micro-sprinkler irrigation systems. Surface irrigation was applied as per conventional farmer practice. Pressurized irrigation treatments consisted of drip irrigation for sugarcane rows and micro-sprinkler irrigation for intercrops. Irrigation scheduling was based on crop water requirements to maintain optimum soil moisture conditions throughout the crop growth period. Hybrid pressurized irrigation ensured efficient water distribution within the root zone and minimized water losses due to evaporation and deep percolation. Micro sprinklers were set up for the intercrops, and inline drip systems will be placed between the sugarcane rows.

### 2.5. Growth Attributes 

Growth parameters of sugarcane were recorded at different crop growth stages in plant crop, ratoon-I and ratoon-II from randomly selected representative plants from each plot. Number of tillers (×10^3^ ha^−1^) was recorded at 120 days after planting (DAP) by counting total tillers from a fixed row length in each plot and converting values to a hectare basis. Plant height (cm) was measured at the maturity stage from ground level to the top visible dewlap leaf using a measuring scale from ten randomly selected plants in each plot, and the mean value was computed. Leaf area index (LAI) was recorded at 240 DAP using a leaf area meter by measuring leaf area of sampled plants and dividing by ground area occupied by the crop. LAI was calculated as the ratio of total leaf area per plant to ground area covered by the plant. Dry matter accumulation (t ha^−1^) was determined at harvest stage by collecting aboveground plant samples from a representative area of each plot (8 m × 5.4 m: 43.2 m^2^). Plant samples were oven-dried at 65 ± 5 °C until constant weight was obtained, and dry weight was expressed on a hectare basis. Growth observations were recorded following standard agronomic procedures widely used in field crop experiments.

### 2.6. Yield Attributes

Yield-contributing parameters were recorded at the harvest stage from ten randomly selected representative plants from the net plot area in each treatment. Cane diameter (cm) was measured at the middle portion of the internode using a digital vernier caliper, and the mean value was calculated. The number of millable canes (×10^3^ ha^−1^) was determined by counting the total number of harvestable canes present in the net plot area and converted to a hectare basis. Only healthy and millable canes suitable for harvest were considered. Single cane weight (g plant^−1^) was recorded by weighing randomly selected millable canes using an electronic balance, and the average cane weight was computed. Yield attribute observations were recorded following standard procedures commonly adopted in sugarcane agronomy experiments.

### 2.7. Crop Yield

Crop yield of sugarcane and intercrops was recorded from the net plot area at physiological maturity and expressed as tonnes per hectare (t ha^−1^) as indicated in Table 3. Canes from each net plot area were harvested manually, cleaned and weighed using a field balance. Yield was converted to a hectare basis based on net harvested area. Yield of intercrops was recorded separately from respective plots. French bean yield was recorded during 2017–2018, 2018–2019 and 2019–2020 cropping seasons, whereas green gram yield was recorded during 2018, 2019 and 2020 seasons. Sugarcane yield was recorded separately for plant crop, ratoon-I and ratoon-II to evaluate the sustainability of the production system under different irrigation and intercropping treatments. Crop yield estimation followed standard agronomic methods used in field experiments.

### 2.8. Juice Quality Parameters

Sugarcane juice quality parameters were determined at harvest from randomly selected representative millable canes collected from each plot. Cane samples were crushed using a power-operated cane crusher, and fresh juice was analyzed for quality characteristics following standard procedures.

Brix (%) was measured using a digital refractometer, which indicates the total soluble solids present in the juice. Pol (%) representing sucrose content was determined using a polarimeter according to the standard sugarcane juice analysis procedure. Juice purity (%) was calculated as the ratio of Pol to Brix.

Commercial cane sugar (CCS %) was calculated using the standard formula:

CCS (%) = [Pol (%) − (Brix (%) − Pol (%) × 0.4] × 0.73

Sugar yield (t ha^−1^) was calculated by multiplying cane yield with CCS percentage:

Sugar yield (t ha^−1^) = Cane yield (t ha^−1^) × CCS (%)/100

All juice quality parameters were determined following standard analytical procedures described in the cane sugar handbook and widely adopted sugarcane quality evaluation protocols [8].

### 2.9. System Productivity

System productivity of the sugarcane-based intercropping system was evaluated using cane equivalent yield (CEY) to account for productivity of component crops on a common yield basis. Cane equivalent yield was calculated based on prevailing market prices of sugarcane and intercrops during the respective crop years.

CEY (t ha^−1^) was calculated using the following equation:

CEY = Yield of intercrop × (Market price of intercrop/Market price of sugarcane) + Yield of sugarcane

The cane equivalent yield allows comparison of productivity of intercropping treatments with sole sugarcane system by converting yield of intercrops into equivalent sugarcane yield on economic basis. This approach provides a comprehensive assessment of biological productivity and economic advantage of diversified cropping systems.

The method of equivalent yield estimation has been widely used for evaluating productivity of intercropping systems [9,10].

### 2.10. Water Use Efficiency

Water use efficiency (WUE) was calculated to evaluate irrigation efficiency in terms of crop production per unit of water applied.

WUE (kg ha^−1^ cm^−1^) was calculated as:

WUE = Cane equivalent yield (kg ha^−1^)/Total water applied (cm)

Total water applied included irrigation water and effective rainfall received during the crop growth period (Data available in Supplementary Table S3. Water productivity was also expressed in economic terms as net return per unit water applied. Similar approaches for estimation of water use efficiency have been widely used in agronomic and irrigation studies [11,12].

### 2.11. Soil Sampling and Analysis

#### 2.11.1. Soil Microbial Activity

Fresh soil samples were collected at harvest of the ratoon II (Data available in Supplementary Table S4) to determine soil microbial biomass carbon (SMBC) and other soil quality indicators, following the procedure described by [13].

#### 2.11.2. Soil Carbon Fractions and Carbon Lability

Soil carbon fractions were determined following the modified [14] procedure described by [15,16]. Oxidizable carbon (Cox) and its different fractions were estimated by sequential oxidation of 1.0 g of soil using H_2_SO_4_ at different concentrations. Very labile carbon (Fraction I; VL) was determined after oxidation with 6.0 M H_2_SO_4_. Labile carbon (Fraction II; L) was calculated as the difference between the oxidizable carbon measured after oxidation with 9.0 M and 6.0 M H_2_SO_4_. Less labile carbon (Fraction III; LL) was calculated as the difference between the oxidizable carbon measured after oxidation with 12.0 M and 9.0 M H_2_SO_4_. Non-labile carbon (Fraction IV; NLL) was estimated from the organic carbon remaining after oxidation with 12.0 M H_2_SO_4_ in relation to total organic carbon (TOC).

Total organic carbon (TOC) was determined using the wet oxidation method [17]. The carbon lability index (LI) was calculated according to [18] as:

$$\mathrm{LI}=\left[\left(\frac{C_{\mathrm{VL}}}{\mathrm{TOC}}\right)\times 3+\left(\frac{C_{L}}{\mathrm{TOC}}\right)\times 2+\left(\frac{C_{\mathrm{LL}}}{\mathrm{TOC}}\right)\times 1\right]$$

where C_VL_, C_L_, and C_LL_ represent very labile, labile, and less labile carbon fractions, respectively, and TOC represents total organic carbon.

#### 2.11.3. Soil Microbial Biomass Carbon

Soil microbial biomass carbon (SMBC) was estimated using the chloroform fumigation–extraction method of [19]. Fresh soil samples were collected at each sampling stage. One portion of the soil was used for the determination of soil moisture content, while two sets of samples were maintained under refrigerated conditions as non-fumigated controls for 24 h. The remaining two sets were subjected to chloroform fumigation.

For fumigation, ethanol-free chloroform was placed in a 50 mL beaker inside a vacuum desiccator containing the soil samples. The samples were fumigated under vacuum for 24 h. After fumigation, the vacuum was released, and the fumigated samples were removed from the desiccator. Both fumigated and non-fumigated soil samples were separately transferred into conical flasks and extracted by shaking with 25 mL of K_2_SO_4_ solution for 30 min. The extracts were subsequently analyzed by titration with ferrous ammonium sulfate using ferroin as the indicator.

Microbial biomass carbon was calculated from the difference between the fumigated and non-fumigated samples using a conversion factor (K_C_) of 0.45:

$$\mathrm{SMBC}=\frac{F_{C}}{K_{C}}$$

where F_C_ represents the difference in carbon released from the fumigated and non-fumigated soil samples, and K_C_ is the extraction-conversion factor, taken as 0.45.

#### 2.11.4. Carbon Indices

Carbon indices were computed to assess the effect of treatments on soil carbon dynamics. Carbon pool index (CPI) was calculated as the ratio of total organic carbon in treatment to that of control. Carbon lability index (CLI) was computed as the ratio of labile to non-labile carbon in treatment relative to control. Carbon management index (CMI) was derived as the product of CPI and CLI multiplied by 100. Soil organic carbon stock was estimated using bulk density and sampling depth. These indices provide an integrated assessment of carbon sequestration and soil quality improvement under different management practices. The various carbon indices were calculated [20] as follows:

Carbon Pool Index (CPI)

$$\mathrm{CPI}=\frac{\mathrm{Total}\;\mathrm{Organic}\;\mathrm{Carbon}\;(\mathrm{treatment})}{\mathrm{Total}\;\mathrm{Organic}\;\mathrm{Carbon}\;(\mathrm{Control})}$$

Carbon Lability Index (CLI)

$$\mathrm{CLI}=\frac{\mathrm{Labile}\;\mathrm{Carbon}/\text{Non-labile}\;\mathrm{Carbon}\;(\mathrm{treatment})}{\mathrm{Labile}\;\mathrm{Carbon}/\text{Non-labile}\;\mathrm{Carbon}\;(\mathrm{control})}$$

Carbon Management Index (CMI)

CMI = CPI × CLI × 100

Total Carbon Stock

∆SOC stock = SOC (%) × Bulk density (Mgm^−3^) × Depth (cm)

Lability of SOC

$$\mathrm{SOC}\;\mathrm{lability}=\frac{\mathrm{Labile}\;\mathrm{Carbon}}{\text{Non-labile}\;\mathrm{Carbon}}$$

Samples from Ratoon II were separated into particulate organic matter (POM) using the size-fractionation procedure [20]. Briefly, 10 g of air-dried soil samples were dispersed in 0.5% sodium hexametaphosphate for 18 h on a reciprocal shaker. Each sample was then washed with distilled water through a 63 µm sieve. The fraction retained on the sieve was considered the POM fraction, and the finer fraction that passed through the sieve was considered the MAOM fraction. The POM was dried at 60 °C for 24 h and ground with a ball mill.

### 2.12. Economic Analysis

Economic analysis was carried out using cost of cultivation, gross return, net return and benefit–cost ratio based on prevailing market prices of inputs and produce during respective crop years.

Gross return (₹ ha^−1^) was calculated by multiplying cane equivalent yield with the market price of produce. Net return (₹ ha^−1^) was obtained by subtracting cost of cultivation from gross return, while benefit–cost ratio was calculated as the ratio of net return to cost of cultivation. Similar procedures for economic evaluation of cropping systems have been widely adopted in agronomic studies [21].

### 2.13. Statistical Analysis

Experimental data were analyzed using analysis of variance appropriate for randomized block design to evaluate experimental treatment effects on crop growth, yield, quality parameters and resource-use efficiency. Treatment means were compared using the least significant difference test at the 5% probability level. Standard error of the mean and coefficient of variation were calculated to assess variability between treatments and reliability of experimental results.

Statistical analysis procedures followed standard methods described for agricultural experiments [22].

## 3. Results

### 3.1. Growth Parameters

#### 3.1.1. Tillers Population

Tiller density was significantly influenced by irrigation method and intercropping configuration in the cropping system (Table 2). Drip-irrigated sole sugarcane (T_2_) showed the highest tiller population (174.4 × 10^3^ ha^−1^), which was 18.2% greater than the surface-irrigated sole crop (T_1_). Intercropping with French bean under conventional irrigation (T_3_) reduced tiller density by 14.0% compared with T1. Adoption of pressurized irrigation improved tiller production under intercropping systems. Sugarcane intercropped with French bean and green gram under drip irrigation (T_7_) increased tiller density by 27.1% compared with T_3_. Residue incorporation (T_8_) further enhanced ratoon regeneration, producing 0.4% higher tiller density than T_7_ in ratoon-I and 1.5% higher in ratoon-II. Across crop cycles, treatments receiving drip irrigation maintained consistently higher tiller persistence, indicating improved bud sprouting and ratoon vigour relative to surface irrigation treatments.

#### 3.1.2. Plant Height

Plant height responded positively to improved irrigation efficiency and reduced interspecific competition (Table 2). Drip-irrigated sole sugarcane (T_2_) produced maximum plant height (320 cm), representing a 25.0% increase compared with surface-irrigated sole crop (T_1_). Intercropping under surface irrigation (T_3_–T_5_) reduced plant height by 8–15% relative to the sole crop. Pressurized irrigation alleviated the growth limitation observed under conventional intercropping. Sugarcane intercropped with French bean and green gram under drip irrigation (T_7_) recorded 23.4% greater plant height than T_3_ in plant crop. Residue incorporation (T_8_) further increased plant height by 0.8% compared with T_7_ across ratoon crops. Although plant height declined slightly in ratoon-II relative to plant crop, treatment ranking remained unchanged, with drip irrigation treatments maintaining greater stalk elongation.

#### 3.1.3. Leaf Area Index (LAI)

Leaf area index was significantly affected by irrigation regime and intercropping combination (Table 2). Drip-irrigated sole crop (T_2_) produced the highest LAI (4.36), which was 16.6% higher than surface-irrigated sole crop (T_1_). Surface-irrigated intercropping treatments (T_3_–T_5_) recorded 12–15% lower LAI relative to T_1_, indicating restricted canopy expansion. Pressurized irrigation enhanced canopy development under intercropping. Sugarcane intercropped with French bean and green gram under drip irrigation (T_7_) recorded 21.9% higher LAI compared with T_3_. Residue incorporation (T_8_) further increased LAI by 0.8% across ratoon crops. Higher LAI under drip irrigation treatments indicates improved radiation interception and canopy growth efficiency.

#### 3.1.4. Dry Matter Accumulation

Dry matter production differed significantly between treatments (Table 2). Drip-irrigated sole sugarcane (T_2_) accumulated the maximum biomass (37.3 t ha^−1^), representing 26.4% higher dry matter compared with surface-irrigated sole crop (T_1_). Intercropping with French bean under surface irrigation (T_3_) reduced biomass accumulation by 19.3% relative to T_1_. Pressurized irrigation substantially improved biomass accumulation under intercropping systems. Sugarcane intercropped with French bean and green gram under drip irrigation (T_7_) recorded 42.0% greater dry matter than T_3_. Residue incorporation (T_8_) further improved biomass accumulation by 5–7% compared with T_7_ across crop cycles. Dry matter production decreased slightly in ratoon-II relative to plant crop, but relative treatment differences remained consistent.

### 3.2. Yield Parameters 

#### 3.2.1. Cane Diameter

Cane diameter did not vary significantly between treatments (Table 4). In the plant crop, cane diameter ranged from 2.24 to 2.63 cm. Maximum cane diameter was recorded in T_2_, which were 17.4% higher than T_3_ and 11.9% higher than T_4_. Intercropping treatments T_7_ and T_8_ recorded greater cane diameter compared with conventional intercropping treatments. Residue incorporation increased cane diameter by 3.9–4.5% compared with treatments without residue incorporation. Similar trends were observed in ratoon crops.

#### 3.2.2. Number of Millable Canes

Millable cane population showed significant variation between treatments (Table 4). Drip-irrigated sole crop (T_2_) produced 15.7% greater millable cane density compared with surface-irrigated sole crop (T_1_). Intercropping under conventional irrigation (T_3_–T_5_) reduced millable cane population by 6–9% compared with sole crop. Pressurized irrigation increased millable cane population substantially. Sugarcane intercropped with French bean and green gram under drip irrigation (T_7_) produced 16.6% more millable canes compared with T_3_. Residue incorporation (T_8_) further enhanced millable cane population by 19.5% compared with T_4_ in ratoon-I and 19.8% compared with T_3_ in ratoon-II.

#### 3.2.3. Single Cane Weight

Single cane weight did not show variation between treatments (Table 4). Drip-irrigated sole crop (T_2_) recorded the highest cane weight (937 g plant^−1^), which was 10.4% greater than French bean intercropping under surface irrigation (T_3_). Differences between intercropping treatments remained marginal, indicating that variation in yield was primarily associated with millable cane population rather than individual cane mass.

### 3.3. Crop Productivity

#### 3.3.1. Sugarcane and Intercrop Yield

Sugarcane yield varied significantly between treatments and ranged from 78.7 to 107.3 t ha^−1^ (Table 3). The highest cane yield was recorded in T2 (107.3 t ha^−1^), followed by T_8_ (101.4 t ha^−1^) and T_7_ (99.8 t ha^−1^). Drip irrigation increased cane yield by 19.6% compared with surface irrigation treatment (T_1_). Intercropping treatments combined with pressurized irrigation recorded 24.4–26.3% higher yield compared with conventional intercropping treatments. Residue incorporation improved yield by 1.6–3.4% compared with non-residue treatments, indicating a cumulative beneficial effect of organic matter recycling. In ratoon-I, the highest yield was recorded under T_2_ (102.1 t ha^−1^), followed by T8 (95.4 t ha^−1^). Drip irrigation increased yield by 18.2% compared with conventional irrigation. Residue incorporation treatments maintained higher ratoon productivity, indicating improved soil structure and nutrient availability. In ratoon-II, the highest yield was recorded in T2 (98.4 t ha^−1^), followed by T8 (89.8 t ha^−1^). Yield improvement under pressurized irrigation ranged between 16.7 and 22.0% compared with conventional irrigation. Decline in yield in ratoon crops compared with the plant crop was observed across treatments; however, integrated irrigation and residue incorporation treatments minimized yield reduction, indicating sustainability of the production system.

#### 3.3.2. Intercrop Yield

French bean yield was significantly influenced by irrigation method and intercropping combination (Table 3). Yield ranged from 1.03 to 1.79 t ha^−1^. The highest French bean yield was recorded in T_8_ (1.79 t ha^−1^), followed by T_6_ (1.58 t ha^−1^) and T_7_ (1.57 t ha^−1^). Hybrid irrigation treatments recorded 28.7–42.6% higher yield compared with surface irrigation treatments. Residue incorporation improved French bean yield by 10.5% (Table 4) compared with T_7,_ indicating a positive effect of organic residue recycling on soil nutrient supply. Lower yield observed in T_3_ (1.03 t ha^−1^) may be attributed to greater interspecific competition and limited moisture availability under surface irrigation. Green gram yield ranged from 0.71 to 1.14 t ha^−1^ across treatments. The highest yield was recorded in T_8_ (1.14 t ha^−1^), which was 60.6% higher than T_4_ (Table 3). Residue incorporation significantly improved green gram productivity due to improved soil organic matter and nutrient availability. Higher yield under hybrid irrigation treatments indicated efficient water distribution and improved root growth environment.

### 3.4. Juice Quality Parameters and Yield

#### 3.4.1. Brix Percentage

Brix percentage showed marginal variation between irrigation and intercropping treatments across crop cycles (Table 5). Drip-irrigated sole sugarcane (T_2_) recorded the highest Brix value (19.7%) in the plant crop, representing 5.3% higher than sugarcane intercropped with French bean and green gram under surface irrigation (T_4_).

Surface-irrigated intercropping treatments (T_3_–T_5_) exhibited relatively lower Brix values (18.2–18.8%), whereas drip-based intercropping treatments (T_6_–T_8_) maintained comparatively higher Brix (19.3–19.8%). In ratoon-I, drip-irrigated treatments produced 5.6% higher Brix compared with conventional intercropping treatments. Across crop cycles, variation in Brix remained within a narrow range (18.2–19.8%), indicating limited influence of intercropping systems on total soluble solids accumulation.

#### 3.4.2. Pol Percentage

Pol percentage differed slightly between treatments but remained statistically comparable across crop cycles (Table 5). Drip-irrigated sole crop (T_2_) recorded the highest pol content (17.30%) in the plant crop, which was 6.3% higher than sugarcane intercropped with French bean and green gram under surface irrigation (T_4_). Pressurized irrigation treatments (T_6_–T_7_) maintained relatively higher pol content (17.22–17.53%) compared with surface-irrigated intercropping treatments (16.06–16.35%). Residue incorporation treatment under surface irrigation (T_5_) recorded comparatively lower pol values across crop cycles. Differences in pol content between plant crop and ratoon crops remained minimal, suggesting stable sucrose accumulation irrespective of cropping system.

#### 3.4.3. Purity Percentage

Juice purity percentage showed limited variation across irrigation and intercropping treatments (Table 5). Purity values ranged from 86.6 to 89.9% across crop cycles. Drip-irrigated intercropping with French bean under micro-sprinkler irrigation (T_6_) recorded the highest purity (89.9%) in ratoon-II, representing a 2.4% improvement over surface-irrigated intercropping with residue incorporation (T_5_). Surface-irrigated treatments (T_3_–T_5_) exhibited purity values between 86.6 and 88.6%, whereas drip-irrigated treatments (T_6_–T_8_) recorded relatively higher purity values (87.5–89.9%). Overall, juice purity remained statistically similar between treatments, indicating negligible effect of intercropping and irrigation system on sucrose content in juice.

#### 3.4.4. Commercial Cane Sugar (CCS %)

Commercial cane sugar (CCS %) showed relatively narrow variation between irrigation and intercropping treatments across plant and ratoon crops. Drip-irrigated sole sugarcane (T_2_) recorded comparatively higher CCS values than surface-irrigated sole crop (T_1_), indicating improved sucrose recovery efficiency under uniform soil moisture availability. Between intercropping treatments, sugarcane intercropped with French bean and green gram under drip irrigation (T_7_) maintained a higher CCS percentage than conventional intercropping systems (T_3_–T_5_). Residue incorporation (T_8_) resulted in marginal improvement (1.5–3.0%) in CCS over the non-residue intercropping treatment (T_7_), suggesting a beneficial effect of organic residue recycling on sucrose accumulation as indicated in Table 6. Across crop cycles, CCS values remained relatively stable; indicating that intercropping did not adversely affect juice quality and sucrose recovery potential. A slight decline in CCS was observed in ratoon-II compared with plant crop, but relative treatment superiority remained unchanged (Table 6).

#### 3.4.5. Yield

Sugar yield, being a function of cane yield and CCS percentage, varied significantly between treatments (Figure 2a). Drip-irrigated sole sugarcane (T_2_) recorded the highest sugar yield, reflecting the combined advantage of higher cane productivity and improved juice quality traits. Sugar yield under T_2_ was approximately 20–22% higher than the surface-irrigated sole crop (T_1_). Between intercropping treatments, sugarcane intercropped with French bean and green gram under drip irrigation with residue incorporation (T_8_) recorded higher sugar yield compared with surface-irrigated intercropping treatments (T_3_–T_5_). Sugar yield under T_8_ was approximately 25–30% greater than the conventional intercropping treatment (T_3_), indicating improved assimilates partitioning and sucrose accumulation efficiency under pressurized irrigation. Ratoon crops showed slightly lower sugar yield compared with the plant crop; however, pressurized irrigation treatments maintained relatively higher sugar productivity across crop cycles. Improved sugar yield under drip irrigation treatments reflects the positive influence of improved water availability on sink development and sucrose translocation.

#### 3.4.6. Cane Equivalent Yield

Cane equivalent yield differed significantly between treatments due to combined productivity of sugarcane and intercrops (Table 7). Sugarcane intercropped with French bean and green gram under drip irrigation with residue incorporation (T_8_) recorded the highest CEY (154.0, 153.2 and 159.1 t ha^−1^ in plant crop, ratoon-I and ratoon-II, respectively), representing 71.7%, 77.3% and 98.1% higher CEY compared with sole sugarcane under surface irrigation (T_1_). Drip-irrigated intercropping without residue incorporation (T_7_) also recorded high CEY (151.3–149.7 t ha^−1^), which was 68–86% higher than T_1_. Residue incorporation improved CEY by 1.8–9.5% compared with the corresponding treatment without residue incorporation (T_7_). Under surface irrigation, intercropping with French bean and green gram (T_4_) improved CEY by 38.2%, 34.8% and 48.7% compared with sole sugarcane (T_1_), indicating the advantage of a multi-crop system over monocropping. Overall, CEY clearly demonstrated the superiority of drip-irrigated legume intercropping systems in improving land productivity.

### 3.5. Water Use Efficiency and Water Productivity

Water use efficiency (WUE) was significantly affected by irrigation method and cropping system across crop cycles (Table 8). Drip-irrigated sole sugarcane (T_2_) recorded the highest WUE (1529.14 kg ha^−1^ cm), which was 92.4% higher than sole sugarcane under surface irrigation (T_1_; 794.65 kg ha^−1^ cm).

Between intercropping treatments, sugarcane intercropped with French bean under drip irrigation (T_6_) recorded 68.6% higher WUE than sugarcane + French bean under surface irrigation (T_3_). Similarly, sugarcane intercropped with French bean and green gram under drip irrigation (T_7_) recorded 0.5% higher WUE than surface-irrigated intercropping with both legumes (T_4_) in the plant crop. Residue incorporation slightly improved WUE. Sugarcane intercropped with French bean and green gram with residue incorporation (T_8_) recorded 1.8% higher WUE than the treatment without residue incorporation (T_7_) in the plant crop and 6.4% higher WUE in ratoon-II (Table 8).

Water productivity also showed significant variation across treatments. Drip-irrigated sole sugarcane (T_2_) recorded the highest water productivity (82.05 ₹ m^−3^), which was 793% higher than surface-irrigated sole sugarcane (T1; 9.19 Rs m^−3^). Between intercropping treatments, the drip-irrigated system with residue incorporation (T_8_) recorded 53.5% higher water productivity than surface-irrigated intercropping with residue incorporation (T_5_). Overall, pressurized irrigation significantly improved water productivity (Figure 2b) and WUE compared with the conventional irrigation system.

### 3.6. Economic Performance

Economic analysis revealed significant variation in profitability between irrigation and intercropping treatments (Table 9). Sugarcane intercropped with French bean and green gram under drip irrigation with residue incorporation (T_8_) recorded the highest net return (₹315.0 × 10^3^ ha^−1^ in ratoon-II), which was 106.4% higher than sole sugarcane under surface irrigation (T_1_). In the plant crop, net return under T8 (₹290.42 × 10^3^ ha^−1^) was 69.8% higher than T_1_ (₹171.02 × 10^3^ ha^−1^). Similarly, sugarcane intercropped with both legumes under drip irrigation (T_7_) recorded 64.9% higher net return than T_1_. Drip-irrigated sole sugarcane (T_2_) recorded 25.3% higher net return than surface-irrigated sole crop (T_1_). Under surface irrigation, intercropping with both legumes (T_4_) increased net return by 35.6% compared with sole cropping (Table 9).

Benefit–cost ratio ranged from 1.30 to 2.13. The highest B: C ratio was recorded under drip-irrigated sole sugarcane (T_2_; 2.13), followed by drip-irrigated intercropping with residue incorporation (T_8_; 1.82) as indicated in Table 9. Overall results indicated that integration of legumes with pressurized irrigation significantly improved profitability compared with the sole sugarcane system.

### 3.7. Soil Physical Properties

Soil physical properties showed slight improvement under residue incorporation treatments after three cropping cycles (Table 10). Bulk density decreased from 1.42 g cm^−3^ under sole sugarcane (T_1_) to 1.37 g cm^−3^ under sugarcane + French bean + green gram with residue incorporation (T_8_), representing a 3.5% reduction in soil compaction. Residue incorporation under surface irrigation (T_5_) also reduced bulk density by 2.8% compared with T_1_. Reduction in bulk density indicates improvement in soil structure due to the addition of organic residues and enhanced root activity under the intercropping system.

### 3.8. Soil Chemical Properties

Soil chemical properties were moderately influenced by high-intensity intercropping and residue incorporation after three cropping cycles (Table 10). Integration of legumes along with residue recycling improved soil fertility parameters compared with the sole sugarcane system. Soil pH showed a slight reduction from 8.38 under T_1_ to 8.21 under T_8_, indicating marginal improvement in soil reaction towards the neutral range. However, differences in soil pH between treatments were statistically non-significant, suggesting the buffering capacity of calcareous soil. Electrical conductivity decreased from 0.32 dS m^−1^ in T_1_ to 0.20 dS m^−1^ in T_8_, showing a 37.5% reduction in soluble salt concentration, indicating improvement in soil chemical environment under residue recycling. A similar trend was observed under T_5,_ where EC decreased by 31.3% compared with T_1_. Soil organic carbon showed consistent improvement under intercropping treatments. Organic carbon content increased from 0.46% under sole sugarcane with surface irrigation (T_1_) to 0.58% under sugarcane + French bean + green gram with residue incorporation (T_8_), representing a 26.1% increase after three crop cycles. Residue incorporation under surface irrigation (T_5_) also improved organic carbon to 0.55%, indicating a 19.6% increase compared with T_1_. Drip-irrigated intercropping without residue incorporation (T_7_) recorded 0.53% organic carbon, which was 15.2% higher than the sole cropping system (Table 10).

Available nitrogen showed gradual improvement and increased by 8.0% from 250 kg ha^−1^ in T_1_ to 270 kg ha^−1^ in T_8_. Residue incorporation under surface irrigation (T_5_) also improved available nitrogen by 6.0% compared with T_1_. Drip-irrigated intercropping without residue incorporation (T_7_) recorded 262 kg ha^−1^ available N, showing a 4.8% increase over T_1_. Available phosphorus improved noticeably under intercropping systems. Available P increased from 10.9 kg ha^−1^ in T_1_ to 13.6 kg ha^−1^ in T_8_, representing a 24.8% increase. Treatment T_5_ also recorded 12.8 kg ha^−1^ available P, indicating a 17.4% increase compared with T_1_. Improvement in available P may be attributed to organic acid production during residue decomposition and enhanced microbial activity. Available potassium increased from 100 kg ha^−1^ under sole sugarcane (T_1_) to 110 kg ha^−1^ under residue-incorporated intercropping treatment (T_8_), representing a 10.0% increase. Residue incorporation under surface irrigation (T_5_) also improved available K by 7.0% compared with T_1_ (Table 10). Overall results indicate that legume intercropping combined with residue incorporation improved soil nutrient availability and soil organic carbon status compared with the sole sugarcane system.

### 3.9. Soil Organic Carbon Dynamics

#### 3.9.1. Soil Organic Carbon Fractions

Soil organic carbon fractions were significantly influenced by high-intensity intercropping and residue incorporation after three cropping cycles (Table 11).

Very labile carbon increased significantly under intercropping treatments. Sugarcane + French bean + green gram with residue incorporation (T_8_) recorded the highest very labile SOC (3.74 g kg^−1^) at 0–15 cm depth, which was 70.0% higher than sole sugarcane under surface irrigation (T_1_; 2.20 g kg^−1^). At sub-surface depth (15–30 cm), very labile SOC increased by 61.5% under T_8_ compared with T_1_. Residue incorporation under surface irrigation (T_5_) also increased very labile carbon by 58.2% compared with T_1_ (Table 11).

Labile SOC increased markedly due to legume integration. Treatment T_8_ recorded 2.48 g kg^−1^ labile carbon at 0–15 cm depth, representing a 134% increase over T_1_ (1.06 g kg^−1^). A similar improvement was observed under T_5_ (119% increase). Subsurface soil also showed improvement, where T8 recorded 154% higher labile carbon than T_1_ (Table 11).

Less labile carbon increased from 0.55 g kg^−1^ in T_1_ to 1.32 g kg^−1^ in T_8_ at 0–15 cm depth, indicating a 140% increase. At 15–30 cm depth, less labile carbon increased by 46.0% under T_8_ compared with T_1_.

The non-labile carbon fraction showed moderate improvement under the integrated cropping system. T_8_ recorded 10.25 g kg^−1^ non-labile SOC, which was 29.6% higher than T_1_. Residue incorporation under surface irrigation (T_5_) increased the stable carbon fraction by 24.3% compared with T_1_ (Table 11). Overall results indicate that intercropping with legumes and residue incorporation improved both active and stable SOC pools.

#### 3.9.2. Total Soil Organic Carbon

Total soil organic carbon (SOC) was significantly influenced by intercropping and residue incorporation after three cropping cycles (Figure 2c). SOC content ranged from 11.75 to 17.81 g kg^−1^ at 0–15 cm soil depth across treatments. Sugarcane + French bean + green gram with residue incorporation (T_8_) recorded the highest SOC (17.81 g kg^−1^), which was 51.6% higher than sole sugarcane under surface irrigation (T_1_; 11.75 g kg^−1^). Residue incorporation under surface irrigation (T_5_) also improved SOC to 16.86 g kg^−1^, representing a 43.5% increase over T_1_. Drip-irrigated intercropping without residue incorporation (T_7_) recorded 14.72 g kg^−1^ SOC, which was 25.3% higher than T_1_, indicating a positive contribution of legume integration even without residue recycling.

#### 3.9.3. Particulate Organic Matter (POM)

Particulate organic matter (POM) increased significantly under intercropping and residue incorporation treatments (Table 12). Sugarcane + French bean + green gram with residue incorporation (T_8_) recorded the highest POM (3.62 g kg^−1^ at 0–15 cm), which was 84.7% higher than sole sugarcane under surface irrigation (T_1_; 1.96 g kg^−1^). Residue incorporation under surface irrigation (T_5_) also increased POM to 3.48 g kg^−1^, representing a 77.6% increase compared with T_1_.

Sub-surface POM (15–30 cm depth) increased from 0.93 g kg^−1^ in T_1_ to 1.46 g kg^−1^ in T_8_, indicating a 57.0% increase due to residue incorporation and root biomass contribution. The increase in POM indicates improvement in the active carbon fraction and greater stabilization of organic matter under the legume intercropping system.

#### 3.9.4. Total Soil Inorganic Carbon (SIC) and Total Carbon

Total soil inorganic carbon (SIC) did not show significant variation between treatments after three cropping cycles (Table 12). SIC values ranged from 22.44 to 25.59 g kg^−1^ at 0–15 cm soil depth. The highest SIC was observed under sugarcane + French bean with drip irrigation (T_6_; 25.59 g kg^−1^), which was 14.0% higher than sole sugarcane under surface irrigation (T_1_; 22.44 g kg^−1^). However, the differences between treatments were statistically non-significant, indicating that intercropping and residue incorporation had limited influence on the inorganic carbon fraction in the short term. Total carbon (sum of organic and inorganic carbon) varied significantly between treatments. Highest total carbon was recorded under sugarcane + French bean + green gram with residue incorporation (T8; 42.74 g kg^−1^), which was 25.0% higher than sole sugarcane under surface irrigation (T1; 34.19 g kg^−1^). Residue incorporation under surface irrigation (T_5_) also increased total carbon to 40.80 g kg^−1^, representing a 19.3% increase over T_1_. Drip-irrigated intercropping without residue incorporation (T_7_) recorded 39.21 g kg^−1^ total carbon, which was 14.7% higher than T_1_, indicating the contribution of legume integration towards carbon accumulation. The increase in total carbon under intercropping treatments was mainly attributed to improvement in the organic carbon fraction through residue recycling and enhanced root biomass contribution.

#### 3.9.5. Soil Microbial Biomass Carbon (SMBC)

Soil microbial biomass carbon showed significant improvement under legume intercropping and residue incorporation treatments (Table 12). The highest SMBC was recorded under sugarcane + French bean + green gram with residue incorporation (T_8_; 292.5 mg kg^−1^), which was 22.6% higher than sole sugarcane under surface irrigation (T_1_; 238.7 mg kg^−1^). Residue incorporation under surface irrigation (T_5_) also increased SMBC to 288.7 mg kg^−1^, representing a 20.9% increase over T_1_. Drip-irrigated intercropping without residue incorporation (T_7_) improved SMBC by 10.3% compared with T_1_, indicating a beneficial effect of legume integration on microbial activity. The increase in SMBC under residue incorporation treatments indicates enhanced microbial activity due to increased availability of organic substrates and improved soil micro-environment.

#### 3.9.6. Soil Carbon Indices

Carbon indices were significantly influenced by irrigation methods, intercropping and residue incorporation (Figure 3). The carbon pool index (CPI) ranged from 1.00 in T_1_ to 1.52 in T_8_, indicating a 52% increase in carbon accumulation over control (Figure 3b). Similarly, carbon lability index (CLI) varied from 1.00 to 1.54, suggesting a higher proportion of labile carbon fractions under integrated treatments (Figure 3d). Carbon management index (CMI), which integrates both carbon quantity and quality, was highest in T_8_ (234), followed by T_5_ (213), demonstrating substantial improvement in soil carbon dynamics due to residue incorporation and legume intercropping (Figure 3c). SOC lability also increased from 0.385 in T1 to 0.592 in T_8_, indicating enhanced active carbon pools (Figure 3a). Soil organic carbon stock ranged from 24.9 to 36.6 Mg ha^−1^ (Data available in Supplementary Table S5), with significantly higher values recorded under T_8_ and T_5_, reflecting improved carbon sequestration under intensified cropping systems (Figure 3e).

### 3.10. Irrigation Depth, Effective Rainfall and Soil Moisture Contribution

The irrigation depth was 48 cm under solitary sugarcane with surface irrigation (T1); however, intercropping with French bean (T3) lowered the irrigation depth by 8.33% (44 cm), as depicted in Table 13. The inclusion of green gram in the intercropping system (T4 and T5) lowered the irrigation depth to 32 cm, a 33.33% reduction over T1. Effective rainfall was consistent across all surface-irrigated treatments, measuring 62.7, 71.7, and 115.9 cm for the plant crop, first ratoon, and second ratoon, respectively. Surface-irrigated treatments resulted in minimal variation in soil moisture (about ±1% during plant crops and ≤3.6% for ratoon crops). Drip-irrigated treatments (T2, T6, T7, and T8), on the other hand, had a significantly lower soil moisture contribution than T1 across the three crop cycles, ranging from 23.77 to 27.35%. Between all treatments, T4 (2.19 cm) contributed the most soil moisture during the plant crop, which was 0.46% greater than T1, while T8 (1.60 cm) contributed the least, representing a 26.61% decrease over T1. Similar patterns were found in ratoon crops (Table 13).

## 4. Discussion

The present study demonstrated that hybrid pressurized irrigation significantly improved crop growth parameters, including tiller population, plant height, leaf area index and dry matter accumulation compared with conventional surface irrigation. Improved growth performance under drip irrigation can be attributed to uniform distribution of soil moisture within the root zone, which maintains favorable soil water potential for a longer duration [23]. Frequent application of small quantities of water under pressurized irrigation reduces moisture fluctuation and prevents alternate wetting and drying cycles that adversely affect root activity and nutrient uptake efficiency. Improved soil moisture regime enhances cell division, elongation and metabolic activity, resulting in better vegetative growth and canopy development [24,25].

Higher tiller population observed under pressurized irrigation indicates reduced physiological stress during the formative growth stage of sugarcane. Adequate moisture availability during the early growth period promotes meristematic activity and improves tiller emergence and survival. Increased plant height under drip irrigation may be associated with improved stomatal conductance and higher photosynthetic efficiency due to continuous availability of soil moisture [26]. Improved leaf area index indicates enhanced canopy architecture and greater interception of photosynthetically active radiation, which ultimately increases biomass production. Higher dry matter accumulation under pressurized irrigation suggests efficient conversion of intercepted radiation into plant biomass, indicating improved source strength under optimum soil moisture conditions [23]. Improvement in growth parameters subsequently enhanced yield attributes including cane diameter, millable cane population and single cane weight. Yield attributes represent sink capacity of crop and are strongly influenced by soil moisture availability during grand growth stage [24]. Continuous availability of moisture under drip irrigation promotes internodal elongation and improves assimilate partitioning towards storage tissues, resulting in increased cane girth and higher individual cane weight. Higher millable cane population under pressurized irrigation also indicates improved tiller survival and reduced mortality under favorable soil moisture environment.

Intercropping of sugarcane with legumes such as French bean and green gram improved system productivity through complementary utilization of growth resources [23]. Root exudates released by legume crops enhance microbial activity and improve nutrient solubilization in the rhizosphere. Differences in rooting pattern between sugarcane and legumes allow efficient exploitation of soil nutrients from different soil depths, thereby reducing interspecific competition and improving overall nutrient uptake efficiency [25]. Improved intercrop yield under pressurized irrigation indicates that adequate soil moisture availability plays a critical role in achieving higher productivity in intercropping systems.

Higher cane equivalent yield observed under legume intercropping treatments indicates the advantage of diversified cropping systems over monoculture. Intercropping systems improve biological efficiency through temporal and spatial niche differentiation, enabling component crops to utilize environmental resources more efficiently. Inclusion of legumes enhances productivity, thereby improving resource use efficiency and sustainability of the production system [26]. Residue incorporation of green gram further enhanced growth and yield performance of sugarcane crop. Addition of crop residues contributes organic carbon and essential nutrients, which improve soil aggregation and enhance soil physical condition. Decomposition of residues releases nutrients gradually, which ensures continuous nutrient supply throughout the crop growth period [27]. Incorporation of legume residues improves soil organic matter content and promotes formation of stable soil aggregates, which enhances infiltration rate, water-holding capacity and root proliferation [28]. Legume residues generally possess a lower C:N ratio, which promotes faster decomposition and rapid mineralization of nutrients. Enhanced availability of nutrients under residue incorporation treatments improves nutrient uptake efficiency and contributes to improved vegetative growth and biomass accumulation. Decline in yield from plant crop to ratoon crops is commonly observed due to deterioration of soil structure and gradual depletion of available nutrients. However, integration of pressurized irrigation with residue incorporation helped maintain higher productivity of ratoon crops compared with conventional practices. Continuous supply of soil moisture under drip irrigation promotes rapid activation of underground buds and improves ratoon establishment. Improved soil organic matter content under residue incorporation treatments enhances soil structural stability and reduces soil compaction, which facilitates better root growth and nutrient uptake in ratoon crops. Maintenance of higher cane weight in ratoon crops indicates efficient assimilate partitioning under improved soil fertility and moisture availability. Juice quality parameters including Brix, pol and CCS (Table 2) were slightly improved under pressurized irrigation treatments. Adequate soil moisture availability during the maturity stage improves carbohydrate metabolism and enhances sucrose accumulation in cane stalk. Balanced vegetative growth under optimum soil moisture regime supports efficient translocation of photosynthates towards storage tissues, resulting in improved juice quality [29]. Higher CCS percentage observed under integrated treatments indicates improved sugar recovery potential, which ultimately contributes to higher sugar yield.

Soil carbon pools improved significantly under legume intercropping and residue incorporation treatments. An increase in total SOC and particulate organic matter indicates greater addition of organic residues through crop biomass and root turnover. Improvement in labile carbon fractions suggests enhanced microbial substrate availability, while an increase in non-labile carbon indicates stabilization of carbon in more recalcitrant pools. Enhanced microbial biomass carbon under residue incorporation treatments indicates improvement in soil biological activity and nutrient transformation processes [30,31]. Increased microbial activity accelerates decomposition and mineralization processes, which improve nutrient availability to crop plants. Improvement in soil chemical properties under legume intercropping treatments indicates a positive influence of integrated nutrient cycling [32]. Improvement in available phosphorus and potassium suggests enhanced nutrient solubilization due to microbial activity and production of organic acids during decomposition [33]. Slight reduction in soil bulk density under residue incorporation treatments indicates improvement in soil structure and porosity, which facilitates better root growth and water movement in soil. The higher values of CPI (Figure 3b), CLI (Figure 3d) and CMI (Figure 3c) under T_8_ and T_5_ treatments can be attributed to increased organic matter input through residue incorporation and diversified cropping systems. Legume-based intercropping contributed to greater biomass addition and improved nitrogen availability, thereby enhancing microbial activity and carbon turnover [32]. The increase in SOC lability indicates a greater proportion of labile carbon fractions, which play a crucial role in nutrient cycling [34,35]. The increase in SOC under intercropping treatments indicates enhanced carbon input through crop residues [36].

Higher CMI values (>200) under T_8_ clearly indicate restoration and improvement of soil carbon status. Pressurized irrigation systems further supported carbon stabilization by maintaining optimal soil moisture conditions, thereby promoting microbial processes and carbon sequestration.

Water use efficiency improved substantially under pressurized irrigation treatments due to reduction in conveyance losses, evaporation losses and deep percolation losses. Frequent application of small quantities of water under drip irrigation maintains optimum soil moisture in the root zone, which improves nutrient diffusion and uptake efficiency [37]. Higher water productivity observed under integrated treatments indicates improved economic return per unit water consumed, highlighting the suitability of a pressurized irrigation system for water-limited environments [38].

The economic analysis revealed that the integrated irrigation and intercropping treatments resulted in significantly higher profitability compared to other treatments. This improvement in economic returns can be attributed to the additional yield contributed by the intercrop component, which enhanced total system income without a proportionate increase in production costs [39,40]. Although the initial capital investment required for the installation of pressurized irrigation systems is comparatively higher than conventional methods, this cost is offset over the long term through improved crop yield, greater water use efficiency [41] and enhanced overall system productivity [42], thereby ensuring favorable cost–benefit outcomes. Furthermore, incorporation of crop residues contributed to improved soil fertility [43], which is likely to reduce dependency on external fertilizer inputs [44,45] over successive cropping cycles, thus enhancing the long-term economic sustainability of the system. In addition, the adoption of a diversified cropping system was found to mitigate production risk by generating supplementary income sources, thereby strengthening the resilience and stability of the farming system against climatic variability and market fluctuations.

Our results indicate that integration of pressurized irrigation with legume intercropping and residue incorporation improves crop productivity, soil health and resource use efficiency, thereby contributing to sustainable intensification of sugarcane-based cropping systems.

## 5. Conclusions

The present study showed significant improvement in growth, yield attributes and productivity of sugarcane under hybrid pressurized irrigation compared with conventional surface irrigation. Drip irrigation recorded the highest cane yield (107.3 t ha^−1^ in plant crop and 98.4 t ha^−1^ in ratoon-II), which was about 19–24% higher than conventional irrigation treatments. Improved crop growth was reflected in higher millable cane population (115.6 × 10^3^ ha^−1^) and greater single cane weight (937 g plant^−1^) under the pressurized irrigation system. Integration of legume intercropping improved system productivity, where maximum cane equivalent yield was recorded (154.0 t ha^−1^ in plant crop) under sugarcane (PR) with drip irrigation + French bean (3 rows)—green gram (3 rows) with micro sprinkler irrigation to both the intercrops + residue incorporation of green gram, showing about 71–98% higher productivity compared with the sole cropping system. Residue incorporation further improved soil organic carbon (17.81 g kg^−1^) and enhanced sustainability of ratoon productivity. Pressurized irrigation significantly enhanced water use efficiency, with maximum WUE observed under T_2_ (1529 kg ha^−1^ cm^−1^), which was about 92% higher than conventional irrigation. Economic analysis also revealed higher profitability under integrated treatments, with maximum net return recorded under sugarcane (PR) with drip irrigation + French bean (3 rows)—green gram (3 rows) with micro sprinkler irrigation to both the intercrops + residue incorporation of green gram (₹315.0 × 10^3^ ha^−1^ in ratoon-II). The highest CPI, CLI and CMI values under sugarcane (PR) with drip irrigation + French bean (3 rows)—green gram (3 rows) with micro sprinkler irrigation to both the intercrops + residue incorporation of green gram indicate enhanced carbon sequestration and improved soil health. These findings highlight the potential of integrated management practices for sustaining soil fertility and long-term productivity of sugarcane-based systems. The integration of hybrid pressurized irrigation, legume intercropping and residue incorporation proved to be an efficient strategy for improving productivity, water use efficiency and economic returns, thereby providing a sustainable approach for intensification of sugarcane-based production systems under limited water availability conditions.

## Figures and Tables

**Figure 1:**
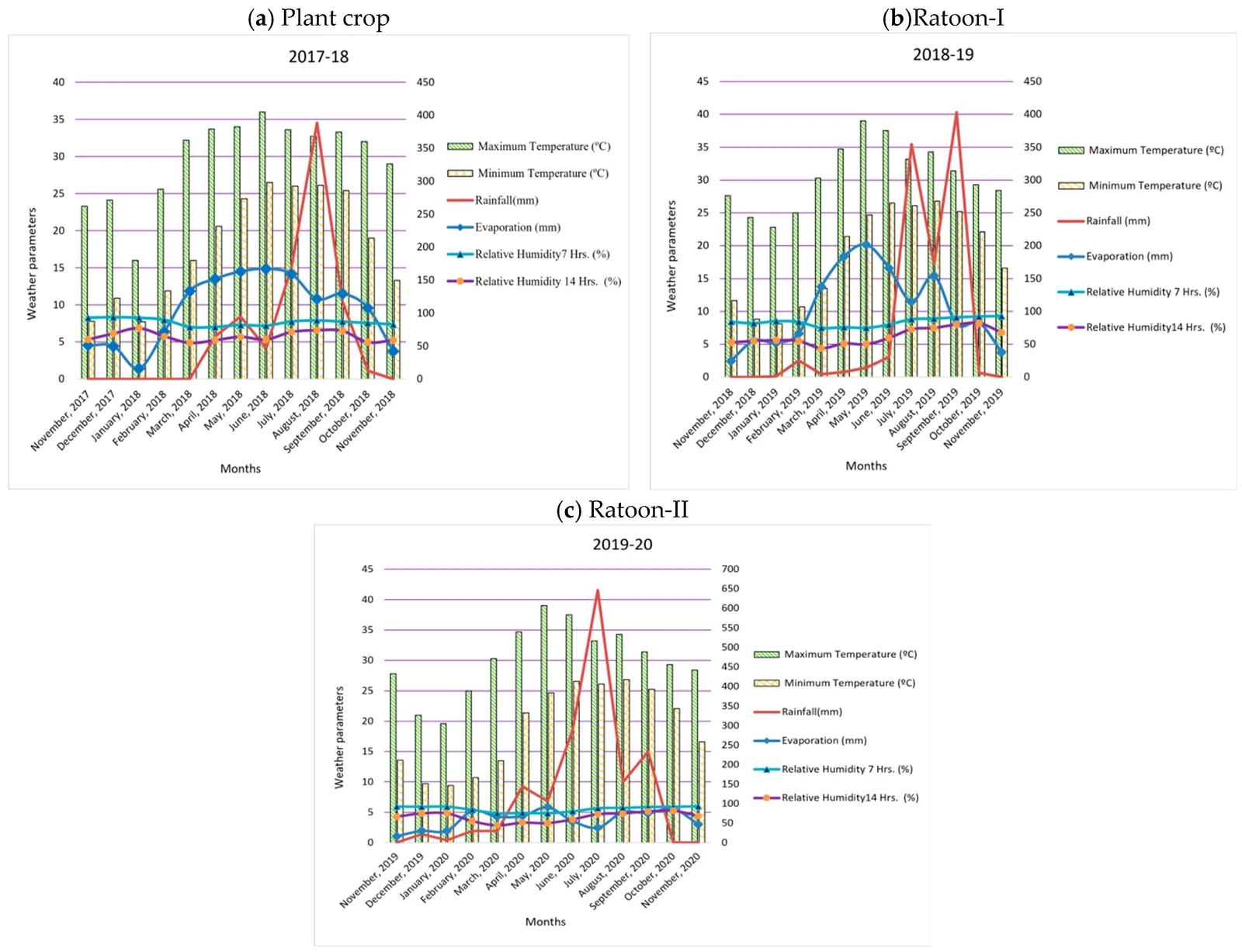
Weather parameters during experimental years (2017–2018, 2018–2019, 2019–2020) in plant-ratoon-ratoon cycles.

**Figure 2:**
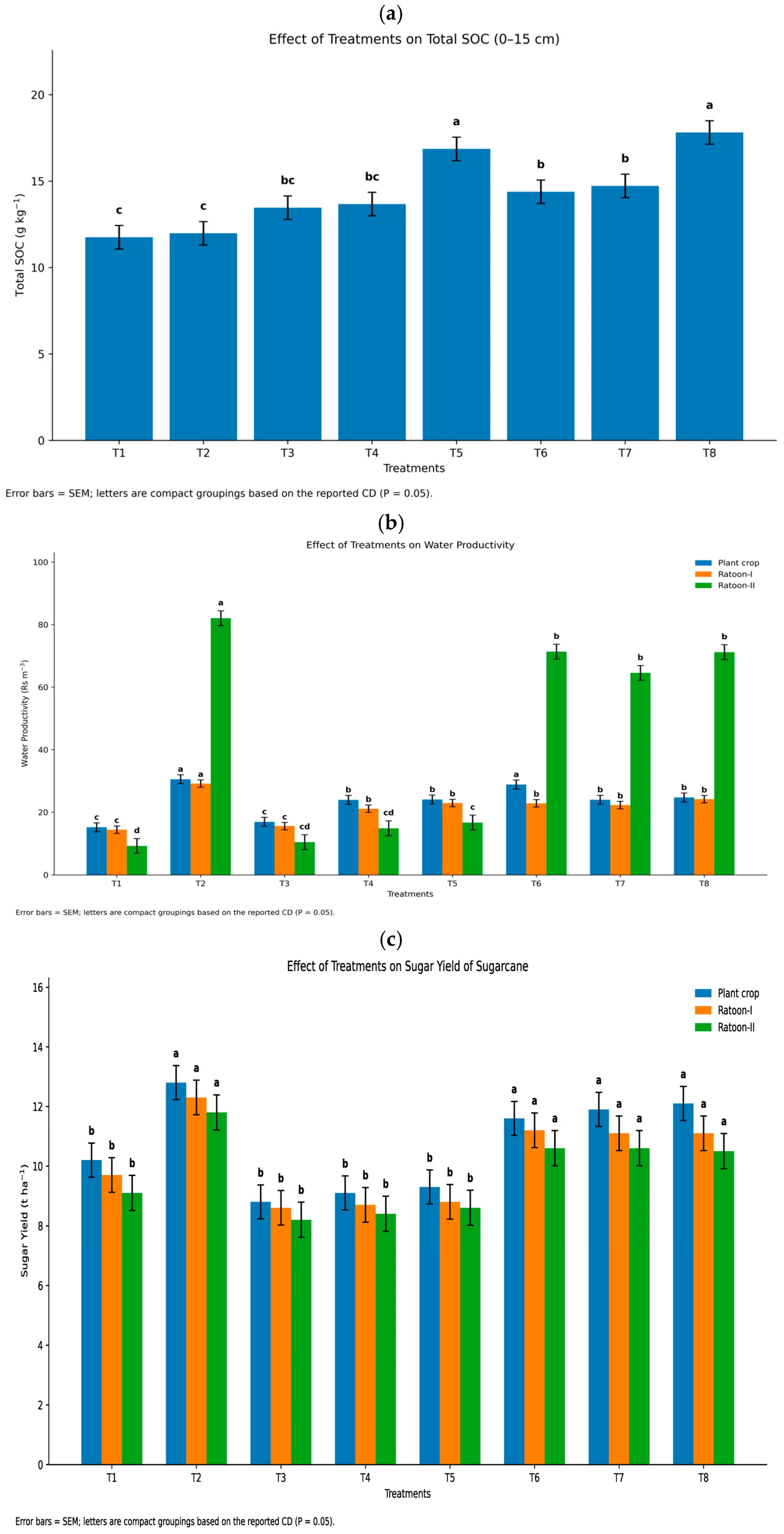
Effect of hybrid pressurized irrigation on (**a**) total SOC, (**b**) water productivity and (**c**) sugar yield of sugarcane under a sugarcane-based intercropping system (Different letters in lowercase showed significant variation as per DMRT).

**Figure 3:**
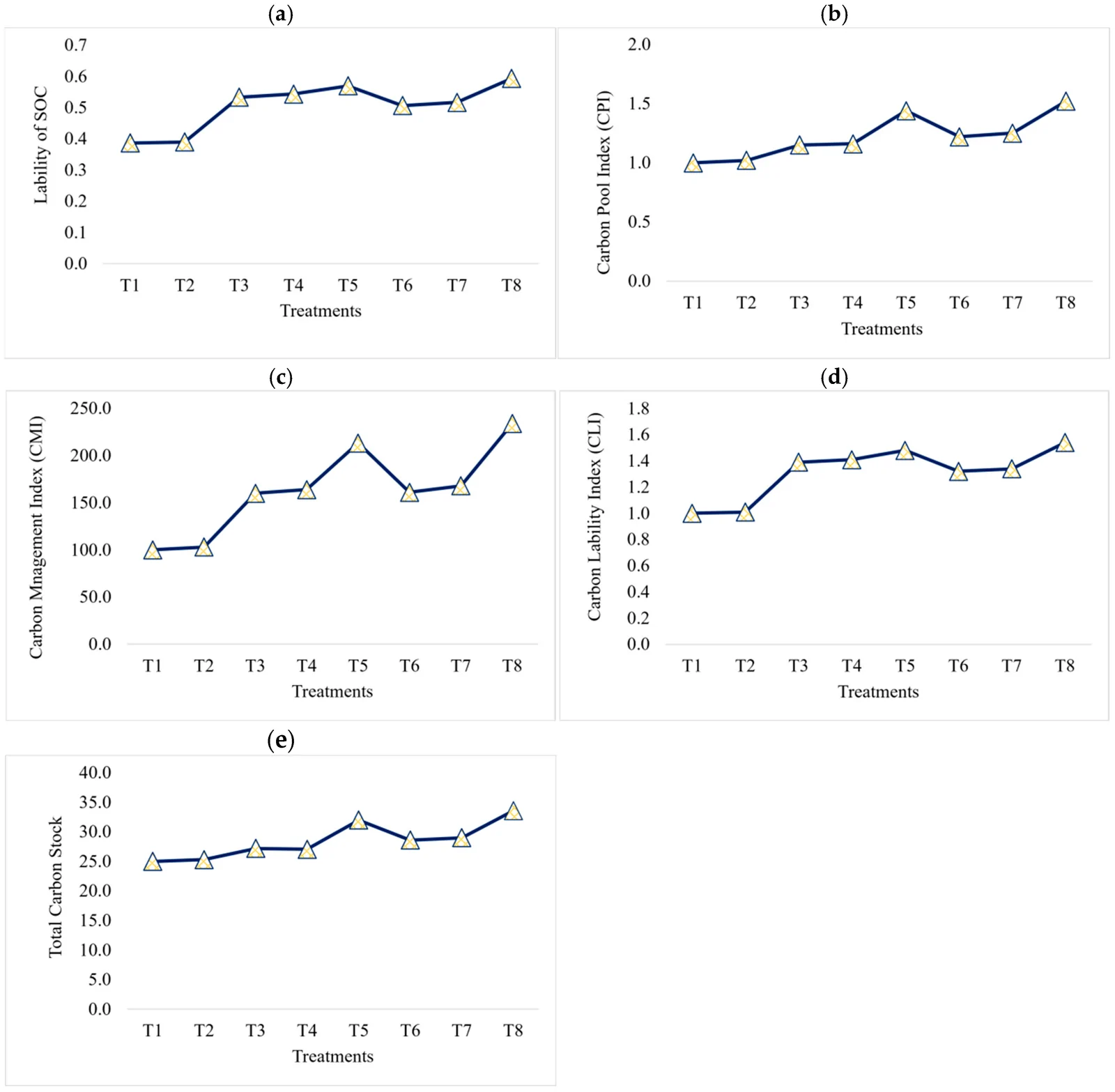
Effect of hybrid pressurized irrigation on (**a**) lability of SOC, (**b**) carbon pool index (CPI), (**c**) carbon management index (CMI), (**d**) carbon lability index (CLI) and (**e**) total carbon stock (TOC) under a sugarcane-based intercropping system.

**Table 1:** Treatment details.

Treatment	Cropping System	Irrigation System	Residue Incorporation
_1_	Sole sugarcane	Surface irrigation	No
T_2_	Sole sugarcane	Drip irrigation	No
T_3_	Sugarcane + French bean (3 rows)	Surface irrigation	No
T_4_	Sugarcane + French bean—green gram (3 rows)	Surface irrigation	No
T_5_	Sugarcane + French bean—green gram (3 rows)	Surface irrigation	Green gram residue incorporated
T_6_	Sugarcane + French bean (3 rows)	Drip irrigation (sugarcane) + micro-sprinkler (intercrops)	No
T_7_	Sugarcane + French bean—green gram (3 rows)	Drip irrigation (sugarcane) + micro-sprinkler (intercrops)	No
T_8_	Sugarcane + French bean—green gram (3 rows)	Drip irrigation (sugarcane) + micro-sprinkler (intercrops)	Green gram residue incorporated after two pickings

**Table 2:** Effect of hybrid pressurized irrigation on tillers, plant height, LAI and dry matter accumulation under a sugarcane-based intercropping system.

Treatment		Tillers at 120 DAP (×10^3^ ha^−1^)			Plant Height (cm)			LAI at 240 DAP			Dry Matter Accumulation (t ha^−1^)		
		Plant Crop	Ratoon-I	Ratoon-II	Plant Crop	Ratoon-I	Ratoon-II	Plant Crop	Ratoon-I	Ratoon-II	Plant Crop	Ratoon-I	Ratoon-II
T_1_	Sole sugarcane (PR) with surface irrigation	147.5 ^bc^ ± 8.88	154.8 ^bc^ ± 8.09	140.5 ^bc^ ± 9.11	256 ^bc^ ± 15.9	244.7 ^bc^ ± 13.98	238.5 ^bc^ ± 16.30	3.74 ^bc^ ± 0.21	3.78 ^bc^ ± 0.21	3.75 ^bc^ ± 0.21	29.5 ^bc^ ± 1.88	28.2 ^bc^ ± 1.50	25.7 ^bc^ ± 1.39
T_2_	Sole sugarcane (PR) with drip irrigation	174.4 ^a^ ± 8.79	174.3 ^a^ ± 8.12	168.5 ^a^ ± 9.13	320 ^a^ ± 16.23	299.3 ^a^ ± 13.88	301.2 ^a^ ± 16.33	4.36 ^a^ ± 0.21	4.38 ^a^ ± 0.21	4.25 ^a^ ± 0.20	37.3 ^a^ ± 1.60	34.1 ^a^ ± 1.49	33.8 ^a^ ± 1.39
T_3_	Sugarcane (PR) + French bean (3 rows) with surface irrigation/farmers’ practice	126.8 ^d^ ± 8.77	129.5 ^d^ ± 8.08	119.6 ^d^ ± 9.10	236 ^c^ ± 16.14	224.3 ^c^ ± 13.95	226.4 ^c^ ± 16.29	3.26 ^c^ ± 0.21	3.22 ^c^ ± 0.21	3.35 ^c^ ± 0.21	23.8 ^d^ ± 1.64	23.4 ^d^ ± 1.47	22.1 ^d^ ± 1.39
T_4_	Sugarcane (PR) + French bean—green gram (3 rows) with surface irrigation	134.2 ^cd^ ± 8.83	133.6 ^cd^ ± 8.09	125.8 ^cd^ ± 9.11	245 ^bc^ ± 16.23	233.0 ^bc^ ± 14.03	231.4 ^bc^ ± 16.30	3.56 ^bc^ ± 0.21	3.76 ^bc^ ± 0.21	3.58 ^bc^ ± 0.21	24.2 ^cd^ ± 1.75	24.3 ^cd^ ± 1.52	23.2 ^cd^ ± 1.39
T_5_	T_4_ + Residue incorporation of green gram	134.3 ^cd^ ± 8.82	152.2 ^bc^ ± 8.12	140.9 ^bc^ ± 9.12	249 ^bc^ ± 16.42	239.3 ^bc^ ± 13.96	241.5 ^bc^ ± 16.30	3.57 ^bc^ ± 0.21	3.68 ^bc^ ± 0.21	3.61 ^bc^ ± 0.21	25.9 ^cd^ ± 1.72	24.7 ^cd^ ± 1.51	23.5 ^cd^ ± 1.39
T_6_	Sugarcane (PR) with drip irrigation + French bean (3 rows) with micro-sprinkler irrigation	159.8 ^ab^ ± 8.81	165.0 ^ab^ ± 8.11	153.4 ^ab^ ± 9.12	294 ^ab^ ± 16.23	288.0 ^ab^ ± 13.99	292.2 ^ab^ ± 16.32	3.94 ^ab^ ± 0.21	3.96 ^ab^ ± 0.21	3.82 ^ab^ ± 0.21	32.3 ^ab^ ± 1.69	30.4 ^ab^ ± 1.50	27.9 ^ab^ ± 1.39
T_7_	Sugarcane (PR) with drip irrigation + French bean (3 rows)—green gram (3 rows) with micro-sprinkler irrigation to both the intercrops	161.2 ^ab^ ± 8.84	166.3 ^ab^ ± 8.10	157.8 ^ab^ ± 9.12	303 ^a^ ± 16.23	294.7 ^a^ ± 14.02	296.8 ^a^ ± 16.32	4.04 ^a^ ± 0.21	4.02 ^a^ ± 0.21	3.92 ^ab^ ± 0.21	33.8 ^ab^ ± 1.68	30.7 ^ab^ ± 1.49	28.6 ^ab^ ± 1.39
T_8_	T_7_ + Residue incorporation of green gram	162.6 ^ab^ ± 8.82	167.0 ^ab^ ± 8.13	160.2 ^ab^ ± 9.13	307 ^a^ ± 16.23	297.0 ^a^ ± 14.05	299.0 ^a^ ± 16.33	4.07 ^a^ ± 0.21	4.06 ^a^ ± 0.21	3.95 ^a^ ± 0.21	35.7 ^ab^ ± 1.68	32.4 ^ab^ ± 1.52	30.2 ^ab^ ± 1.39
	SEm ±	8.15	7.77	8.91	15.6	10.29	16.34	0.194	0.201	0.201	1.62	1.45	1.34
	CD (*p* = 0.05)	24.7	23.6	27.0	47	40.3	49.6	0.59	0.61	0.61	4.9	4.4	4.1
	CV (%)	9.4	8.7	10.6	10	8.7	10.6	8.82	9.01	9.22	9.3	8.8	8.6

PR: Paired row; LAI: Leaf Area Index; DAP: Days after planting; SEm: Standard error of the mean; CD: Critical difference; CV: Coefficient of variation; Different letters in lowercase showed significant variation as per Duncan Multiple Range Test (DMRT); NS: Non-significant.

**Table 3:** Effect of treatment on yield of intercrops and sugarcane under sugarcane-based intercropping system.

Treatment		Yield (t ha^−1^)								
		French Bean			Green Gram			Sugarcane		
		2017–2018	2018–2019	2019–2020	2018	2019	2020	Plant Crop	Ratoon-I	Ratoon-II
T_1_	Sole sugarcane (PR) with surface irrigation	–	–	–	–	–	–	89.7 ± 5.31 ^bc^	86.4 ± 4.56 ^b^	80.3 ± 5.20 ^b^
T_2_	Sole sugarcane (PR) with drip irrigation	–	–	–	–	–	–	107.3 ± 5.52 ^a^	102.1 ± 4.59 ^a^	98.4 ± 5.23 ^a^
T_3_	Sugarcane (PR) + French bean (3 rows) with surface irrigation/farmers’ practice	1.28 ± 0.087	1.09 ± 0.084	1.03 ± 0.075	–	–	–	78.7 ± 5.32 ^c^	77.3 ± 4.56 ^c^	72.9 ± 5.30 ^b^
T_4_	Sugarcane (PR) + French bean—green gram (3 rows) with surface irrigation	1.36 ± 0.087	1.19 ± 0.078	1.14 ± 0.076	0.77 ± 0.046	0.71 ± 0.046	0.73 ± 0.05	81.6 ± 5.41 ^c^	78.6 ± 4.60 ^c^	74.9 ± 5.34 ^b^
T_5_	T_4_ + Residue incorporation of green gram	1.29 ± 0.087	1.28 ± 0.081	1.30 ± 0.075	0.76 ± 0.046	0.85 ± 0.046	0.83 ± 0.05	83.5 ± 5.33 ^c^	80.1 ± 4.59 ^c^	77.4 ± 5.26 ^b^
T_6_	Sugarcane (PR) with drip irrigation + French bean (3 rows) with micro-sprinkler irrigation	1.63 ± 0.087	1.52 ± 0.084	1.58 ± 0.076	–	–	–	97.4 ± 5.41 ^ab^	93.1 ± 4.58 ^ab^	86.4 ± 5.20 ^ab^
T_7_	Sugarcane (PR) with drip irrigation + French bean (3 rows)—green gram (3 rows) with micro-sprinkler irrigation to both intercrops	1.61 ± 0.087	1.60 ± 0.078	1.57 ± 0.075	0.97 ± 0.046	1.01 ± 0.046	1.04 ± 0.05	99.8 ± 5.39 ^a^	93.3 ± 4.59 ^ab^	87.4 ± 5.30 ^ab^
T_8_	T_7_ + Residue incorporation of green gram	1.68 ± 0.093	1.75 ± 0.087	1.79 ± 0.076	0.96 ± 0.046	1.14 ± 0.006	1.12 ± 0.05	101.4 ± 5.42 ^a^	95.4 ± 4.60 ^a^	89.8 ± 5.40 ^ab^
	SEm ±	–	–	–	–	–	–	5.12	4.45	5.21
	CD (*p* = 0.05)	–	–	–	–	–	–	15.5	13.5	15.8
	CV (%)	–	–	–	–	–	–	9.6	8.7	10.8

PR: Paired row: SEm: Standard error of the mean; CD: Critical difference; CV: Coefficient of variation; Different letters in lowercase showed significant variation; NS: Non-significant.

**Table 4:** Effect of treatment on cane diameter, millable canes and single cane weight of sugarcane plant crop, ratoon-I and ratoon-II under sugarcane-based intercropping system.

Treatment		Cane Diameter (cm)			Millable Canes (×10^3^ ha^−1^)			Single Cane Weight (g Plant^−1^)		
		Plant Crop	Ratoon-I	Ratoon-II	Plant Crop	Ratoon-I	Ratoon-II	Plant Crop	Ratoon-I	Ratoon-II
T_1_	Sole sugarcane (PR) with surface irrigation	2.46 ± 0.156 ^a^	2.30 ± 0.121 ^a^	2.35 ± 0.10 ^a^	99.9 ± 6.09 ^bc^	102.9 ± 5.52 ^bc^	97.2 ± 5.65 ^bc^	901 ± 47.4 ^a^	845 ± 41.53 ^a^	843 ± 43.3 ^a^
T_2_	Sole sugarcane (PR) with drip irrigation	2.63 ± 0.157 ^a^	2.32 ± 0.116 ^a^	2.26 ± 0.10 ^a^	115.6 ± 2.67 ^a^	122.6 ± 5.53 ^a^	119.6 ± 5.61 ^a^	937 ± 46.79 ^a^	836 ± 41.2 ^a^	824 ± 43.18 ^a^
T_3_	Sugarcane (PR) + French bean (3 rows) with surface irrigation/farmers’ practice	2.24 ± 0.156 ^a^	2.22 ± 0.121 ^a^	2.31 ± 0.10 ^a^	92.8 ± 6.26 ^c^	93.8 ± 5.50 ^c^	89.8 ± 5.60 ^c^	849 ± 47.36 ^a^	830 ± 41.47 ^a^	821 ± 43.19 ^a^
T_4_	Sugarcane (PR) + French bean—green gram (3 rows) with surface irrigation	2.35 ± 0.157 ^a^	2.15 ± 0.121 ^a^	2.12 ± 0.10 ^a^	93.4 ± 6.20 ^c^	95.2 ± 5.53 ^c^	90.9 ± 5.59 ^c^	877 ± 46.87 ^a^	832 ± 41.54 ^a^	827 ± 43.34 ^a^
T_5_	T_4_ + Residue incorporation of green gram	2.41 ± 0.157 ^a^	2.21 ± 0.116 ^a^	2.22 ± 0.10 ^a^	94.7 ± 6.04 ^c^	96.5 ± 5.52 ^c^	93.1 ± 5.64 ^bc^	885 ± 47.05 ^a^	836 ± 41.46 ^a^	834 ± 43.27 ^a^
T_6_	Sugarcane (PR) with drip irrigation + French bean (3 rows) with micro sprinkler irrigation	2.48 ± 0.156 ^a^	2.36 ± 0.252 ^a^	2.28 ± 0.10 ^a^	107.5 ± 6.10 ^ab^	110.1 ± 5.53 ^ab^	104.7 ± 5.62 ^ab^	908 ± 47.11 ^a^	855 ± 41.33 ^a^	830 ± 43.22 ^a^
T_7_	Sugarcane (PR) with drip irrigation + French bean (3 rows)—green gram (3 rows) with micro-sprinkler irrigation to both the intercrops	2.53 ± 0.158 ^a^	2.31 ± 0.312 ^a^	2.31 ± 0.10 ^a^	108.2 ± 1.45 ^ab^	110.9 ± 5.52 ^ab^	106.2 ± 5.62 ^ab^	926 ± 46.92 ^a^	845 ± 41.56 ^a^	825 ± 43.19 ^a^
T_8_	T_7_ + Residue incorporation of green gram	2.57 ± 0.158 ^a^	2.26 ± 0.116 ^a^	2.18 ± 0.10 ^a^	109.5 ± 0.29 ^ab^	113.8 ± 5.53 ^ab^	108.9 ± 5.63 ^ab^	928 ± 47.08 ^a^	843 ± 41.51 ^a^	825 ± 43.35 ^a^
	SEm ±	0.151	0.150	0.102	4.96	5.28	5.62	45.0	39.7	44.0
	CD (*p* = 0.05)	NS	NS	NS	15.1	16.0	17.1	NS	NS	NS
	CV (%)	10.61	11.48	7.8	8.4	8.7	9.6	9.0	8.0	9.0

PR: Paired row; SEm: Standard error of mean; CD: Critical difference; CV: Coefficient of variation; Different letters in lowercase showed significant variation; NS: Non-significant.

**Table 5:** Effect of hybrid pressurized irrigation on Brix, pol and purity percent of sugarcane.

Treatment		Brix (%)			Pol (%)			Purity (%)		
		Plant Crop	Ratoon-I	Ratoon-II	Plant Crop	Ratoon-I	Ratoon-II	Plant Crop	Ratoon-I	Ratoon-II
T_1_	Sole sugarcane (PR) with surface irrigation	19.0 ± 0.173 ^b^	18.6 ± 0.23 ^b^	18.9 ± 0.29 ^ab^	16.52 ± 0.196 ^b^	16.24 ± 0.16 ^b^	16.49 ± 0.47 ^a^	86.9 ± 1.07 ^a^	87.3 ± 1.04 ^a^	87.2 ± 1.24 ^a^
T_2_	Sole sugarcane (PR) with drip irrigation	19.7 ± 0.173 ^a^	19.8 ± 0.23 ^a^	19.6 ± 0.29 ^a^	17.30 ± 0.201 ^a^	17.45 ± 0.16 ^a^	17.35 ± 0.47 ^a^	87.8 ± 1.04 ^a^	88.1 ± 1.07 ^a^	88.5 ± 1.19 ^a^
T_3_	Sugarcane (PR) + French bean (3 rows) with surface irrigation/farmers’ practice	18.8 ± 0.173 ^b^	18.6 ± 0.23 ^b^	18.4 ± 0.29 ^b^	16.35 ± 0.196 ^b^	16.21 ± 0.16 ^b^	16.21 ± 0.47 ^a^	87.0 ± 1.11 ^a^	87.2 ± 1.04 ^a^	88.1 ± 1.21 ^a^
T_4_	Sugarcane (PR) + French bean—green gram (3 rows) with surface irrigation	18.7 ± 0.173 ^b^	18.5 ± 0.23 ^b^	18.2 ± 0.29 ^b^	16.28 ± 0.196 ^b^	16.13 ± 0.16 ^b^	16.12 ± 0.47 ^a^	87.1 ± 1.05 ^a^	87.2 ± 1.04 ^a^	88.6 ± 1.22 ^a^
T_5_	T_4_ + Residue incorporation of green gram	18.6 ± 0.173 ^b^	18.6 ± 0.23 ^b^	18.3 ± 0.29 ^b^	16.15 ± 0.196 ^b^	16.10 ± 0.16 ^b^	16.06 ± 0.47 ^a^	86.8 ± 1.07 ^a^	86.6 ± 1.04 ^a^	87.8 ± 1.20 ^a^
T_6_	Sugarcane (PR) with drip irrigation + French bean (3 rows) with micro sprinkler irrigation	19.7 ± 0.173 ^a^	19.8 ± 0.23 ^a^	19.5 ± 0.29 ^a^	17.24 ± 0.200 ^a^	17.43 ± 0.16 ^a^	17.53 ± 0.47 ^a^	87.5 ± 1.04 ^a^	88.0 ± 1.06 ^a^	89.9 ± 1.25 ^a^
T_7_	Sugarcane (PR) with drip irrigation + French bean (3 rows)—green gram (3 rows) with micro-sprinkler irrigation to both the intercrops	19.6 ± 0.173 ^a^	19.7 ± 0.23 ^a^	19.6 ± 0.29 ^a^	17.22 ± 0.196 ^a^	17.31 ± 0.16 ^a^	17.47 ± 0.47 ^a^	87.9 ± 1.07 ^a^	87.9 ± 1.04 ^a^	89.1 ± 1.20 ^a^
T_8_	T_7_ + Residue incorporation of green gram	19.4 ± 0.173 ^a^	19.3 ± 0.23 ^ab^	19.2 ± 0.29 ^a^	17.19 ± 0.196 ^a^	16.91 ± 0.16 ^ab^	16.97 ± 0.47 ^a^	88.6 ± 1.06 ^a^	87.6 ± 1.05 ^a^	88.4 ± 1.21 ^a^
	SEm ±	0.167	0.22	0.28	0.190	0.150	0.488	1.01	1.01	1.17
	CD (*p* = 0.05)	0.51	0.70	0.80	0.58	0.46	NS	NS	NS	NS
	CV (%)	1.5	2.0	2.5	1.96	1.56	5.04	2.0	2.0	2.3

Note: PR: Paired row; Means followed by the same letter within a column are not significantly different at *p* = 0.05 according to DMRT; SEm: Standard error of the mean; CD: Critical difference; CV: Coefficient of variation; NS: Non-significant.

**Table 6:** Effect of hybrid pressurized irrigation on CCS percent and sugar yield of sugarcane.

Treatment		CCS (%)		
		Plant Crop	Ratoon-I	Ratoon-II
T_1_	Sole sugarcane (PR) with surface irrigation	11.34 ± 0.269 ^a^	11.17 ± 0.09 ^c^	11.33 ± 0.17 ^bc^
T_2_	Sole sugarcane (PR) with drip irrigation	11.93 ± 0.265 ^a^	12.05 ± 0.09 ^a^	12.01 ± 0.17 ^ab^
T_3_	Sugarcane (PR) + French bean (3 rows) with surface irrigation/farmers’ practice	11.22 ± 0.265 ^a^	11.14 ± 0.09 ^c^	11.20 ± 0.17 ^c^
T_4_	Sugarcane (PR) + French bean—green gram (3 rows) with surface irrigation	11.18 ± 0.272 ^a^	11.08 ± 0.09 ^cd^	11.16 ± 0.19 ^c^
T_5_	T_4_ + Residue incorporation of green gram	11.08 ± 0.271 ^a^	11.02 ± 0.09 ^d^	11.07 ± 0.17 ^c^
T_6_	Sugarcane (PR) with drip irrigation + French bean (3 rows) with micro-sprinkler irrigation	11.87 ± 0.266 ^a^	12.03 ± 0.09 ^a^	12.23 ± 0.17 ^a^
T_7_	Sugarcane (PR) with drip irrigation + French bean (3 rows)—green gram (3 rows) with micro-sprinkler irrigation to both the intercrops	11.88 ± 0.269 ^a^	11.94 ± 0.09 ^a^	12.13 ± 0.17 ^a^
T_8_	T_7_ + Residue incorporation of green gram	11.91 ± 0.264 ^a^	11.64 ± 0.09 ^b^	11.73 ± 0.17 ^bc^
	SEm ±	0.256	0.089	0.164
	CD (*p* = 0.05)	NS	0.27	0.50
	CV (%)	3.84	1.34	2.45

Note: PR: Paired row; Means followed by the same letter within a column are not significantly different at *p* = 0.05 according to DMRT. CCS: Commercial cane sugar percentage; SEm: Standard error of mean; CD: Critical difference; CV: Coefficient of variation; Different letters in lowercase showed significant variation; NS: Non-significant.

**Table 7:** Effect of treatment on yield equivalence and total CEY of sugarcane-based intercropping system.

Treatment		Yield Equivalence (t ha^−1^)								
		French Bean			Green Gram			Total CEY (t ha^−1^)		
		2017–2018	2018–2019	2019–2020	2018	2019	2020	Plant Crop	Ratoon-I	Ratoon-II
T_1_	Sole sugarcane (PR) with surface irrigation	–	–	–	–	–	–	89.7 ± 7.14 ^d^	86.4 ± 6.23 ^d^	80.3 ± 6.18 ^c^
T_2_	Sole sugarcane (PR) with drip irrigation	–	–	–	–	–	–	107.3 ± 7.15 ^cd^	102.1 ± 6.22 ^c^	98.4 ± 6.18 ^c^
T_3_	Sugarcane (PR) + French bean (3 rows) with surface irrigation/farmers’ practice	24.8 ± 1.56	21.1 ± 1.42	23.3 ± 1.79	–	–	–	103.5 ± 7.14 ^cd^	98.4 ± 6.22 ^cd^	96.2 ± 6.19 ^c^
T_4_	Sugarcane (PR) + French bean—green gram (3 rows) with surface irrigation	26.3 ± 1.53	23.0 ± 1.42	25.7 ± 1.83	16.1 ± 0.92	14.9 ± 0.98	18.8 ± 1.27	124.0 ± 7.18 ^bc^	116.5 ± 6.23 ^bc^	119.4 ± 6.20 ^b^
T_5_	T_4_ + Residue incorporation of green gram	25.0 ± 1.58	24.8 ± 1.44	29.4 ± 1.83	15.9 ± 0.95	17.8 ± 0.93	21.4 ± 1.31	124.4 ± 7.16 ^bc^	122.7 ± 6.23 ^b^	128.2 ± 6.18 ^b^
T_6_	Sugarcane (PR) with drip irrigation + French bean (3 rows) with micro-sprinkler irrigation	31.5 ± 1.58	29.4 ± 1.44	35.7 ± 1.74	–	–	–	128.9 ± 7.20 ^b^	122.5 ± 6.23 ^b^	122.1 ± 6.19 ^b^
T_7_	Sugarcane (PR) with drip irrigation + French bean (3 rows)—green gram (3 rows) with micro-sprinkler irrigation to both the intercrops	31.2 ± 1.62	31.0 ± 1.42	35.5 ± 1.79	20.3 ± 0.93	21.2 ± 0.96	26.8 ± 1.28	151.3 ± 7.22 ^a^	145.5 ± 6.23 ^a^	149.7 ± 6.21 ^a^
T_8_	T_7_ + Residue incorporation of green gram	32.5 ± 1.56	33.9 ± 1.47	40.4 ± 1.84	20.1 ± 0.94	23.9 ± 1.13	28.9 ± 1.31	154.0 ± 7.19 ^a^	153.2 ± 6.23 ^a^	159.1 ± 6.20 ^a^
	SEm ±	–	–	–	–	–	–	6.60	5.96	5.99
	CD (*p* = 0.05)	–	–	–	–	–	–	20.0	18.1	18.2
	CV (%)	–	–	–	–	–	–	9.3	8.7	8.7

PR: Paired row: CEY: Cane equivalent yield; SEm: Standard error of mean; CD: Critical difference; CV: Coefficient of variation; Different letters in lowercase showed significant variation according to DMRT; NS: Non-significant Selling price of produce (Rs./t) during 2017–2018 and 2018–2019: Sugarcane @ 3100; French bean @ 60,000; Green gram @ 65,000/; Selling price of produce (Rs./t) during 2019–2020: Sugarcane @ 3100; French bean @ 70,000; Green gram @ 80,000/.

**Table 8:** Water Requirement and water use efficiency of the system as influenced by high-intensity intercropping under pressurized irrigation.

Treatment		Water Requirement (cm)			Water Use Efficiency (kg ha^−1^ cm)		
		Plant Crop	Ratoon-I	Ratoon-II	Plant Crop	Ratoon-I	Ratoon-II
T_1_	Sole sugarcane (PR) with surface irrigation	112.88 ± 5.88 ^a^	121.93 ± 6.93 ^a^	166.13 ± 5.52 ^a^	794.65 ± 72.13 ^a^	708.60 ± 58.90 ^a^	483.40 ± 28.13 ^a^
T_2_	Sole sugarcane (PR) with drip irrigation	70.17 ± 6.22 ^c^	73.85 ± 6.93 ^b^	24.40 ± 5.57 ^b^	1529.14 ± 72.50 ^b^	1382.53 ± 58.93 ^c^	4032.80 ± 128.27 ^a^
T_3_	Sugarcane (PR) + French bean (3 rows) with surface irrigation/farmers’ practice	108.87 ± 6.50 ^a^	117.92 ± 6.93 ^a^	162.08 ± 5.74 ^a^	950.68 ± 72.33 ^a^	834.46 ± 58.91 ^a^	593.50 ± 34.66 ^a^
T_4_	Sugarcane (PR) + French bean—green gram (3 rows) with surface irrigation	96.89 ± 6.44 ^b^	105.91 ± 6.93 ^ab^	150.06 ± 5.72 ^a^	1279.80 ± 72.62 ^b^	1100.00 ± 58.92 ^b^	795.70 ± 45.89 ^a^
T_5_	T_4_ + Residue incorporation of green gram	96.87 ± 6.45 ^b^	105.90 ± 6.93 ^ab^	150.05 ± 5.74 ^a^	1284.20 ± 72.46 ^b^	1158.64 ± 58.92 ^b^	854.40 ± 49.04 ^a^
T_6_	Sugarcane (PR) with drip irrigation + French bean (3 rows) with micro-sprinkler irrigation	80.43 ± 6.24 ^c^	101.09 ± 6.93 ^ab^	31.14 ± 5.72 ^b^	1602.64 ± 72.66 ^b^	1211.79 ± 58.92 ^b^	3921.00 ± 226.84 ^a^
T_7_	Sugarcane (PR) with drip irrigation + French bean (3 rows)—green gram (3 rows) with micro-sprinkler irrigation to both the intercrops	117.65 ± 6.18 ^a^	126.92 ± 6.93 ^a^	44.29 ± 5.63 ^b^	1286.02 ± 72.59 ^b^	1146.39 ± 58.92 ^b^	3380.00 ± 196.44 ^b^
T_8_	T_7_ + Residue incorporation of green gram	117.63 ± 6.51 ^a^	126.90 ± 6.93 ^a^	44.25 ± 5.63 ^b^	1309.19 ± 72.64 ^b^	1207.25 ± 58.92 ^b^	3595.50 ± 207.45 ^b^
	SEm ±	6.048	6.621	5.393	69.492	56.205	128.45
	CD (*p* = 0.05)	18.35	20.09	16.40	210.80	170.50	389.7
	CV (%)	10.46	10.42	9.68	9.59	8.90	10.1

Note: PR: Paired row; Means followed by the same letter within a column are not significantly different at *p* = 0.05 according to DMRT; SEm: Standard error of the mean; CD: Critical difference; CV: Coefficient of variation; NS: Non-significant.

**Table 9:** Economic analysis of treatments under sugarcane-based intercropping system.

Treatment		Cost of Cultivation (×10^3^ Rs ha^−1^)			Gross Returns (×10^3^ Rs ha^−1^)			Net Returns (×10^3^ Rs ha^−1^)			B:C Ratio		
		Plant Crop	Ratoon-I	Ratoon-II	Plant Crop	Ratoon-I	Ratoon-II	Plant Crop	Ratoon-I	Ratoon-II	Plant Crop	Ratoon-I	Ratoon-II
T_1_	Sole sugarcane (PR *) with surface irrigation	107.05	92.18	96.3	278.07 ± 21.9 ^c^	267.8 ± 19.28 ^c^	248.9 ± 20.36 ^c^	171.02 ± 12.6 ^b^	175.62 ± 14.76 ^bc^	152.6 ± 13.17 ^b^	1.60 ± 0.087 ^ab^	1.91 ± 0.11 ^a^	1.58 ± 0.09 ^a^
T_2_	Sole sugarcane (PR) with drip irrigation	118.39	101.22	104.8	332.63 ± 22.26 ^bc^	316.5 ± 19.35 ^b^	305.0 ± 20.28 ^c^	214.24 ± 12.53 ^ab^	215.28 ± 14.78 ^ab^	200.2 ± 13.08 ^ab^	1.81 ± 0.087 ^a^	2.13 ± 0.11 ^a^	1.91 ± 0.09 ^a^
T_3_	Sugarcane (PR) + French bean (3 rows) with surface irrigation/farmers’ practice	136.72	121.85	129.4	320.85 ± 22.04 ^bc^	305.0 ± 19.34 ^bc^	298.2 ± 20.34 ^c^	184.13 ± 12.89 ^ab^	183.15 ± 14.77 ^bc^	168.8 ± 13.23 ^b^	1.35 ± 0.087 ^b^	1.50 ± 0.11 ^b^	1.30 ± 0.08 ^b^
T_4_	Sugarcane (PR) + French bean—green gram (3 rows) with surface irrigation	152.55	137.67	147.3	384.4 ± 21.87 ^ab^	361.2 ± 19.33 ^ab^	370.1 ± 20.40 ^ab^	231.85 ± 12.83 ^a^	223.53 ± 14.77 ^ab^	222.8 ± 13.20 ^a^	1.52 ± 0.09 ^ab^	1.62 ± 0.11 ^ab^	1.51 ± 0.08 ^ab^
T_5_	T_4_ + Residue incorporation of green gram	152.55	137.67	147.3	385.64 ± 22.10 ^ab^	380.4 ± 19.33 ^a^	397.4 ± 20.36 ^ab^	233.09 ± 12.62 ^a^	242.73 ± 14.77 ^a^	250.1 ± 13.27 ^a^	1.53 ± 0.084 ^ab^	1.76 ± 0.11 ^ab^	1.70 ± 0.09 ^ab^
T_6_	Sugarcane (PR) with drip irrigation + French bean (3 rows) with micro-sprinkler irrigation	167.65	149.07	156.3	399.59 ± 22.22 ^ab^	379.8 ± 19.33 ^a^	378.5 ± 20.31 ^ab^	231.94 ± 12.18 ^a^	230.73 ± 14.77 ^ab^	222.2 ± 13.17 ^a^	1.38 ± 0.087 ^b^	1.55 ± 0.11 ^b^	1.42 ± 0.09 ^b^
T_7_	Sugarcane (PR) with drip irrigation + French bean (3 rows)—green gram (3 rows) with micro-sprinkler irrigation to both intercrops	186.98	168.40	178.2	469.03 ± 22.20 ^a^	451.1 ± 19.33 ^a^	464.1 ± 20.33 ^a^	282.05 ± 12.66 ^a^	282.7 ± 14.77 ^a^	285.9 ± 13.13 ^a^	1.51 ± 0.087 ^ab^	1.68 ± 0.11 ^ab^	1.60 ± 0.08 ^ab^
T_8_	T_7_ + Residue incorporation of green gram	186.98	168.40	178.2	477.4 ± 22.25 ^a^	474.9 ± 19.34 ^a^	493.2 ± 20.37 ^a^	290.42 ± 12.63 ^a^	306.5 ± 14.78 ^a^	315.0 ± 13.23 ^a^	1.55 ± 0.087 ^ab^	1.82 ± 0.11 ^ab^	1.77 ± 0.09 ^ab^
	SEm ±	–	–	–	21.243	18.46	19.55	12.163	14.206	12.61	0.084	0.115	0.084
	CD (*p* = 0.05)	–	–	–	64.44	56.0	59.3	36.90	43.09	38.3	0.25	0.32	0.25
	CV (%)	–	–	–	9.66	8.7	9.2	9.17	10.58	9.6	9.48	10.46	9.06

* All economic values are expressed in Indian Rupees (₹); 1 USD ≈ ₹84 (average exchange rate during 2017–2020). Note: Means followed by the same letter within a column are not significantly different at *p* = 0.05 according to DMRT. SEm: Standard error of mean; CD: Critical difference; CV: Coefficient of variation; NS: Non-significant.

**Table 10:** Physical and chemical composition of soil as influenced by 3 years of high-intensity intercropping under pressurized irrigation.

Treatment		pH	EC (dS m^−1^)	Bulk Density (g cm^−3^)	Organic Carbon (%)	Available N (kg ha^−1^)	Available P (kg ha^−1^)	Available K (kg ha^−1^)
T_1_	Sole sugarcane (PR) with surface irrigation	8.38 ± 0.19 ^a^	0.32 ± 0.006 ^a^	1.42 ± 0.04 ^a^	0.46 ± 0.015 ^a^	250 ± 14.43 ^a^	10.9 ± 0.69 ^a^	100 ± 5.77 ^a^
T_2_	Sole sugarcane (PR) with drip irrigation	8.36 ± 0.19 ^a^	0.29 ± 0.006 ^ab^	1.41 ± 0.04 ^a^	0.48 ± 0.015 ^a^	252 ± 14.43 ^a^	11.4 ± 0.69 ^a^	102 ± 5.77 ^a^
T_3_	Sugarcane (PR) + French bean (3 rows) with surface irrigation/farmers’ practice	8.35 ± 0.19 ^a^	0.27 ± 0.006 ^b^	1.41 ± 0.04 ^a^	0.51 ± 0.015 ^a^	255 ± 14.43 ^a^	11.8 ± 0.69 ^a^	103 ± 5.77 ^a^
T_4_	Sugarcane (PR) + French bean—green gram (3 rows) with surface irrigation	8.28 ± 0.19 ^a^	0.24 ± 0.006 ^b^	1.40 ± 0.04 ^a^	0.52 ± 0.015 ^a^	256 ± 14.43 ^a^	12.1 ± 0.69 ^a^	103 ± 6.36 ^a^
T_5_	T_4_ + Residue incorporation of green gram	8.24 ± 0.19 ^a^	0.22 ± 0.006 ^b^	1.38 ± 0.04 ^a^	0.55 ± 0.015 ^a^	265 ± 14.43 ^a^	12.8 ± 0.69 ^a^	107 ± 5.77 ^a^
T_6_	Sugarcane (PR) with drip irrigation + French bean (3 rows) with micro-sprinkler irrigation	8.32 ± 0.19 ^a^	0.25 ± 0.006 ^b^	1.40 ± 0.04 ^a^	0.51 ± 0.015 ^a^	258 ± 14.43 ^a^	11.9 ± 0.69 ^a^	104 ± 5.77 ^a^
T_7_	Sugarcane (PR) with drip irrigation + French bean (3 rows)—green gram (3 rows) with micro-sprinkler irrigation to both the intercrops	8.26 ± 0.19 ^a^	0.24 ± 0.006 ^b^	1.39 ± 0.04 ^a^	0.53 ± 0.015 ^a^	262 ± 14.43 ^a^	12.5 ± 0.69 ^a^	106 ± 5.77 ^a^
T_8_	T_7_ + T_7_ + Residue incorporation of green gram	8.21 ± 0.19 ^a^	0.20 ± 0.006 ^b^	1.37 ± 0.04 ^a^	0.58 ± 0.015 ^a^	270 ± 14.43 ^a^	13.6 ± 0.69 ^a^	110 ± 6.66 ^a^
	SEm ±	0.184	0.006	0.039	0.017	13.9	0.67	5.7
	CD (*p* = 0.05)	NS	0.02	NS	0.05	NS	NS	NS
	CV (%)	3.83	3.80	4.83	5.59	9.0	9.5	9.0

Note: Means followed by the same letter within a column are not significantly different at *p* = 0.05 according to DMRT; SEm: Standard error of the mean; CD: Critical difference; CV: Coefficient of variation; NS: Non-significant.

**Table 11:** Soil organic carbon fractions as influenced by 3 years of high-intensity intercropping under pressurized irrigation.

Treatment		Very Labile SOC (g kg^−1^)		Labile SOC (g kg^−1^)		Less Labile SOC (g kg^−1^)		Non-Labile SOC (g kg^−1^)	
		0–15 cm	15–30 cm	0–15 cm	15–30 cm	0–15 cm	15–30 cm	0–15 cm	15–30 cm
T_1_	Sole sugarcane (PR) with surface irrigation	2.20 ± 0.15 ^d^	1.35 ± 0.08 ^c^	1.06 ± 0.10 ^d^	0.48 ± 0.05 ^c^	0.55 ± 0.05 ^c^	0.63 ± 0.03 ^b^	7.91 ± 0.43 ^c^	6.58 ± 0.33 ^c^
T_2_	Sole sugarcane (PR) with drip irrigation	2.28 ± 0.16 ^d^	1.36 ± 0.08 ^c^	1.09 ± 0.10 ^d^	0.57 ± 0.05 ^c^	0.57 ± 0.05 ^c^	0.61 ± 0.03 ^b^	8.02 ± 0.44 ^c^	6.64 ± 0.33 ^c^
T_3_	Sugarcane (PR) + French bean (3 rows) with surface irrigation/farmers’ practice	2.32 ± 0.16 ^cd^	1.40 ± 0.08 ^c^	1.82 ± 0.10 ^c^	0.89 ± 0.05 ^b^	0.86 ± 0.05 ^b^	0.64 ± 0.03 ^b^	8.45 ± 0.43 ^bc^	6.92 ± 0.33 ^c^
T_4_	Sugarcane (PR) + French bean—green gram (3 rows) with surface irrigation	2.42 ± 0.16 ^c^	1.42 ± 0.08 ^c^	1.84 ± 0.10 ^c^	0.88 ± 0.05 ^b^	0.85 ± 0.05 ^b^	0.59 ± 0.03 ^b^	8.52 ± 0.44 ^bc^	6.86 ± 0.33 ^c^
T_5_	T_4_ + Residue incorporation of green gram	3.48 ± 0.14 ^ab^	1.75 ± 0.08 ^b^	2.32 ± 0.10 ^ab^	1.12 ± 0.05 ^a^	1.21 ± 0.05 ^a^	0.83 ± 0.03 ^a^	9.83 ± 0.45 ^ab^	7.65 ± 0.33 ^b^
T_6_	Sugarcane (PR) with drip irrigation + French bean (3 rows) with micro-sprinkler irrigation	2.55 ± 0.16 ^c^	1.45 ± 0.08 ^c^	1.92 ± 0.10 ^c^	0.97 ± 0.05 ^b^	0.96 ± 0.05 ^b^	0.68 ± 0.03 ^b^	8.94 ± 0.43 ^b^	7.33 ± 0.33 ^b^
T_7_	Sugarcane (PR) with drip irrigation + French bean (3 rows)—green gram (3 rows) with micro-sprinkler irrigation to both the intercrops	2.61 ± 0.16 ^c^	1.52 ± 0.08 ^c^	1.96 ± 0.10 ^c^	1.02 ± 0.05 ^ab^	1.00 ± 0.05 ^b^	0.71 ± 0.03 ^ab^	9.13 ± 0.43 ^b^	7.48 ± 0.33 ^b^
T_8_	T_7_ + Residue incorporation of green gram	3.74 ± 0.14 ^a^	2.18 ± 0.08 ^a^	2.48 ± 0.10 ^a^	1.22 ± 0.05 ^a^	1.32 ± 0.05 ^a^	0.92 ± 0.03 ^a^	10.25 ± 0.44 ^a^	7.72 ± 0.33 ^ab^
	SEm ±	0.150	0.078	0.095	0.045	0.045	0.033	0.419	0.317
	CD (*p* = 0.05)	0.45	0.24	0.29	0.14	0.14	0.10	1.27	0.96
	CV (%)	9.61	8.69	9.04	8.63	8.48	8.24	8.18	7.68

Note: PR: Paired row; Means followed by the same letter within a column are not significantly different at *p* = 0.05 according to DMRT; SEm: Standard error of the mean; CD: Critical difference; CV: Coefficient of variation; NS: Non-significant.

**Table 12:** Status of total SIC, total C, POM and SMBC as influenced by 3 years of high-intensity intercropping under pressurized irrigation.

Treatment		Total SIC (g kg^−1^)	Total C (g kg^−1^)	POM (g kg^−1^)		SMBC (mg kg^−1^)
		0–15 cm	0–15 cm	0–15 cm	15–30 cm	0–15 cm
T_1_	Sole sugarcane (PR) with surface irrigation	22.44 ± 1.20 ^a^	34.19 ± 1.18 ^c^	1.96 ± 0.13 ^c^	0.93 ± 0.06 ^c^	238.7 ± 7.65 ^c^
T_2_	Sole sugarcane (PR) with drip irrigation	22.76 ± 1.21 ^a^	34.74 ± 1.18 ^c^	2.05 ± 0.13 ^c^	0.95 ± 0.06 ^c^	240.8 ± 7.68 ^c^
T_3_	Sugarcane (PR) + French bean (3 rows) with surface irrigation/farmers’ practice	24.90 ± 1.20 ^a^	38.36 ± 1.18 ^b^	2.23 ± 0.13 ^bc^	1.02 ± 0.06 ^bc^	258.6 ± 7.64 ^bc^
T_4_	Sugarcane (PR) + French bean—green gram (3 rows) with surface irrigation	24.87 ± 1.21 ^a^	38.54 ± 1.18 ^b^	2.26 ± 0.13 ^bc^	1.05 ± 0.06 ^bc^	254.2 ± 7.67 ^bc^
T_5_	T_4_ + Residue incorporation of green gram	23.94 ± 1.21 ^a^	40.80 ± 1.18 ^ab^	3.48 ± 0.13 ^a^	1.42 ± 0.06 ^a^	288.7 ± 7.66 ^ab^
T_6_	Sugarcane (PR) with drip irrigation + French bean (3 rows) with micro-sprinkler irrigation	25.59 ± 1.20 ^a^	39.97 ± 1.18 ^ab^	2.45 ± 0.13 ^b^	1.22 ± 0.06 ^ab^	261.5 ± 7.66 ^bc^
T_7_	Sugarcane (PR) with drip irrigation + French bean (3 rows)—green gram (3 rows) with micro-sprinkler irrigation to both the intercrops	24.49 ± 1.20 ^a^	39.21 ± 1.18 ^b^	2.66 ± 0.13 ^b^	1.28 ± 0.06 ^ab^	263.4 ± 7.66 ^bc^
T_8_	T_7_ + Residue incorporation of green gram	24.93 ± 1.20 ^a^	42.74 ± 1.18 ^a^	3.62 ± 0.13 ^a^	1.46 ± 0.06 ^a^	292.5 ± 7.65 ^a^
	SEm ±	1.157	1.133	0.128	0.062	7.29
	CD (*p* = 0.05)	NS	3.44	0.39	0.19	22.1
	CV (%)	8.27	5.09	8.54	9.16	4.8

Note: PR = Paired row; Means followed by the same letter within a column are not significantly different at *p* = 0.05 according to DMRT; SEm: Standard error of the mean; CD: Critical difference; CV: Coefficient of variation; NS: Non-significant.

**Table 13:** Depth of irrigation, effective rainfall and soil moisture contribution as influenced by high-intensity intercropping under pressurized irrigation.

Treatment		Depth of Irrigation (cm)			Effective Rainfall (cm)			Soil Moisture Contribution (cm)		
		Plant Crop	Ratoon-I	Ratoon-II	Plant Crop	Ratoon-I	Ratoon-II	Plant Crop	Ratoon-I	Ratoon-II
T_1_	Sole sugarcane (PR) with surface irrigation	48	48	48	62.7	71.7	115.9	2.18	2.23	2.23
T_2_	Sole sugarcane (PR) with drip irrigation	-	-	-	-	-	-	1.63	1.68	1.70
T_3_	Sugarcane (PR) + French bean (3 rows) with surface irrigation/farmers’ practice	44	44	44	62.7	71.7	115.9	2.17	2.22	2.18
T_4_	Sugarcane (PR) + French bean—green gram (3 rows) with surface irrigation	32	32	32	62.7	71.7	115.9	2.19	2.21	2.16
T_5_	T_4_ + Residue incorporation of green gram	32	32	32	62.7	71.7	115.9	2.17	2.20	2.15
T_6_	Sugarcane (PR) with drip irrigation + French bean (3 rows) with micro-sprinkler irrigation	-	-	-	-	-	-	1.64	1.66	1.68
T_7_	Sugarcane (PR) with drip irrigation + French bean (3 rows)—green gram (3 rows) with micro-sprinkler irrigation to both the intercrops	-	-	-	-	-	-	1.62	1.64	1.67
T_8_	T_7_ + Residue incorporation of green gram	-	-	-	-	-	-	1.60	1.62	1.63

Note: PR: Paired row.

## Data Availability

The original contributions presented in this study are included in the article/Supplementary Material. Further inquiries can be directed to the corresponding authors.

## Supplementary Materials

The following supporting information can be downloaded at: https://www.mdpi.com/article/10.3390/plants15193045/s1.
