# Supplementary material for: Combined Effects of Hybrid Pressurized Irrigation and High-Intensity Legume Intercropping on Productivity, Water Use Efficiency, and Soil Carbon Dynamics in Sugarcane-Based Cropping Systems
Source: Plants (Basel). 2026 Oct 5;15(19):3045. doi: 10.3390/plants15193045 (PMC13667159; doi:10.3390/plants15193045)
Supplement: Supplementary file 1 [file plants-15-03045-s001.zip › plants-4568968-supplementary.pdf]

Supplementary Table S1

**Particulars**	
Net harvested area	8 m × 4.5 m (36 m^2) each plot
Amount of green-gram residue incorporated	6.0 kg/ plot
C concentration/C:N ratio	16:1
Timing	Last week of June (After 2nd pod picking)
Incorporation depth	15 cm

Entire plants were incorporated into the field without being chopped.

Supplementary Table S2.

**Crop Cycle**	**Crop**	**Spacing**	**Date of Planting/Ratooning**	**Harvesting Date**
**Plant**	Sugarcane	30:120	11.11.2017	15.11.2018
	French bean	30 × 10	13.11.2017	22.3.2018
	Green gram	30 × 10	23.3.2018	30.6.2018
**Ratoon I**	Sugarcane	30:120	19.11.2018	15.11.2019
	French bean	30 × 10	20.11.2018	20.3.2019
	Green gram	30 × 10	22.3.2019	17.6.2019
**Ratoon II**	Sugarcane	30:120	18.11.2019	16.11.2020
	French bean	30 × 10	19.11.2019	18.3.2020
	Green gram	30 × 10	20.3.2020	15.6.2020

Gross plot size: Sugarcane: 10 × 9.0 m; French bean: 10× 4.5 m; Green gram: 10× 4.5 m

Net plot size: Sugarcane: 8 × 6.0 m; French bean: 8× 4.5 m; Green gram: 8× 4.5 m

Supplementary Table S3

Year of experiment	Crop	Total seasonal rainfall (mm)	Effective rainfall (mm)
2017-18	Plant crop	895.6	626.9
2018-19	Ratoon I	1024.8	817.4
2019-20	Ratoon II	1655.2	1158.6

This high effective rainfall explains why the **water requirement was significantly lower** during ratoon II

Supplementary Table S4

**Particulars**	
Soil sampling date	Before Sowing/ planting: 05.11.2017 (initial soil sampling)<br>After harvest of Ratoon II crop: 19.11.2020 (Final sampling date)
Sample preparation	As per standard procedures
Soil sampling depth	0-15 and 15- 30 cm
Bulk-density sampling	Core sampler with a core diameter of 3.9 cm and 179.2 cm^3 volume, height 5 cm)
Number of cores/composite samples per plot	Sample were drawn from 4 places in a plot and mixed it to make a composite sample for further analysis

Supplementary Table S5

Treatments		SOC (g kg^{-1})	BD (g cm^{-3})	ΔSOC Stock (Mg ha^{-1})
T_1	Sole sugarcane (PR*) with surface irrigation	11.7	1.42	24.9±1.12^{f}
T_2	Sole sugarcane (PR) with drip irrigation	11.9	1.41	25.2±1.12^{f}
T_3	Sugarcane (PR) + french bean (3 rows) with surface irrigation/ farmers' practice	13.4	1.41	28.3±1.12^{e}
T_4	Sugarcane (PR) + french bean – green gram (3 rows) with surface irrigation	13.6	1.4	28.6±1.12^{e}
T_5	T_4 + Residue incorporation of green gram	16.8	1.38	34.8±1.12^{b}
T_6	Sugarcane (PR) with drip irrigation + french bean (3 rows) with micro sprinkler irrigation	14.3	1.4	30.0±1.12^{d}
T_7	Sugarcane (PR) with drip irrigation + french bean (3 rows) – green gram (3 rows) with micro sprinkler irrigation to both the intercrops	14.7	1.39	30.6±1.12^{c}
T_8	T_7+ Residue incorporation of green gram	17.81	1.37	36.6±1.12^{a}
